# A dual-functional injectable polysaccharide hydrogel incorporating oxygen-carrying nanoemulsion and VEGF for enhancing islet survival and glycemic control in diabetic mice

**DOI:** 10.1093/rb/rbag024

**Published:** 2026-03-05

**Authors:** Yuwei Zhang, Zhaoyu Sun, Miao Liu, Jinxin Pang, Shanshan Huang, Hongyang Liu, Huabin Zheng, Siyu Guan, Jie Li, Yifan Feng, Jiang Ouyang, Tao Xu, Wen Du

**Affiliations:** Key Laboratory of Biological Targeting Diagnosis, Therapy and Rehabilitation of Guangdong Higher Education Institutes, The Fifth Affiliated Hospital & School of Biomedical Engineering, Guangzhou Medical University, Guangzhou, Guangdong 511436, China; Key Laboratory of Biological Targeting Diagnosis, Therapy and Rehabilitation of Guangdong Higher Education Institutes, The Fifth Affiliated Hospital & School of Biomedical Engineering, Guangzhou Medical University, Guangzhou, Guangdong 511436, China; Key Laboratory of Biological Targeting Diagnosis, Therapy and Rehabilitation of Guangdong Higher Education Institutes, The Fifth Affiliated Hospital & School of Biomedical Engineering, Guangzhou Medical University, Guangzhou, Guangdong 511436, China; Key Laboratory of Biological Targeting Diagnosis, Therapy and Rehabilitation of Guangdong Higher Education Institutes, The Fifth Affiliated Hospital & School of Biomedical Engineering, Guangzhou Medical University, Guangzhou, Guangdong 511436, China; Key Laboratory of Biological Targeting Diagnosis, Therapy and Rehabilitation of Guangdong Higher Education Institutes, The Fifth Affiliated Hospital & School of Biomedical Engineering, Guangzhou Medical University, Guangzhou, Guangdong 511436, China; Key Laboratory of Biological Targeting Diagnosis, Therapy and Rehabilitation of Guangdong Higher Education Institutes, The Fifth Affiliated Hospital & School of Biomedical Engineering, Guangzhou Medical University, Guangzhou, Guangdong 511436, China; Key Laboratory of Biological Targeting Diagnosis, Therapy and Rehabilitation of Guangdong Higher Education Institutes, The Fifth Affiliated Hospital & School of Biomedical Engineering, Guangzhou Medical University, Guangzhou, Guangdong 511436, China; Key Laboratory of Biological Targeting Diagnosis, Therapy and Rehabilitation of Guangdong Higher Education Institutes, The Fifth Affiliated Hospital & School of Biomedical Engineering, Guangzhou Medical University, Guangzhou, Guangdong 511436, China; Key Laboratory of Biological Targeting Diagnosis, Therapy and Rehabilitation of Guangdong Higher Education Institutes, The Fifth Affiliated Hospital & School of Biomedical Engineering, Guangzhou Medical University, Guangzhou, Guangdong 511436, China; Key Laboratory of Biological Targeting Diagnosis, Therapy and Rehabilitation of Guangdong Higher Education Institutes, The Fifth Affiliated Hospital & School of Biomedical Engineering, Guangzhou Medical University, Guangzhou, Guangdong 511436, China; Guangzhou Laboratory, Guangzhou, Guangdong 510320, China; Guangzhou Institute of Cancer Research, The Affiliated Cancer Hospital & School of Biomedical Engineering, Guangzhou Medical University, Guangzhou 510180, China; Key Laboratory of Biological Targeting Diagnosis, Therapy and Rehabilitation of Guangdong Higher Education Institutes, The Fifth Affiliated Hospital & School of Biomedical Engineering, Guangzhou Medical University, Guangzhou, Guangdong 511436, China; Guangzhou Laboratory, Guangzhou, Guangdong 510320, China; Key Laboratory of Biological Targeting Diagnosis, Therapy and Rehabilitation of Guangdong Higher Education Institutes, The Fifth Affiliated Hospital & School of Biomedical Engineering, Guangzhou Medical University, Guangzhou, Guangdong 511436, China

**Keywords:** type 1 diabetes, islet transplantation, hydrogel

## Abstract

Type 1 diabetes (T1D) arises from autoimmune destruction of pancreatic β-cells, leading to lifelong dependence on exogenous insulin. Clinical islet transplantation offers a potential cure by restoring endogenous insulin secretion; however, its success is critically limited by poor post-transplant vascularization and oxygen deprivation, which cause over 70% of transplanted islets to undergo early necrosis or apoptosis. To address these challenges, we developed an injectable, biodegradable dual-functional hydrogel composed of oxidized hyaluronic acid (OHA) and carboxymethyl chitosan (CMC). This polysaccharide-based platform co-delivers vascular endothelial growth factor (VEGF) and an oxygen-carrying perfluorotributylamine (PFTBA) nanoemulsion, enabling simultaneous enhancement of angiogenesis and oxygenation within the graft microenvironment. The OHA/CMC hydrogel exhibited excellent biocompatibility, tunable gelation and sustained release of both oxygen and VEGF. *In vitro*, hydrogel-encapsulated islets demonstrated markedly improved resilience under hypoxic stress, exhibiting an 8.6-fold increase in viability after 6 hours and a 4.0-fold enhancement in glucose-stimulated insulin secretion (GSIS) after 48 hours under 0% O_2_. In streptozotocin-induced diabetic mice, transplantation with the OHA/CMC–PFTBA–VEGF hydrogel enabled rapid restoration of endogenous insulin secretion, reversal of severe hyperglycemia, prolonged graft function and enhanced neovascularization at the implant site. Collectively, this multifunctional hydrogel represents a promising and translatable therapeutic platform for improving islet transplantation outcomes in T1D and potentially advancing broader applications in regenerative medicine and complex tissue engineering.

## Graphical abstract

**Graphical Abstract:**
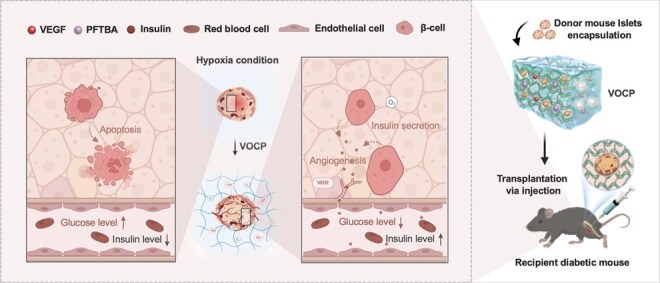

## Introduction

Islet transplantation has emerged as a promising curative treatment for type 1 diabetes (T1D), offering a potential alternative to exogenous insulin injection and whole-pancreas transplantation [1–3]. However, fewer than 15% of recipients remain insulin-independent after 2 years [4]. A major hurdle is the maintenance of graft viability, as 50–60% of transplanted islets are typically lost during the early post-transplant period [5]. This significant loss often requires islets from multiple donors to achieve insulin independence, further exacerbating the already critical shortage of islet donors. The vascularization within islets is denser than that in the surrounding exocrine tissue, leading to a higher oxygen partial pressure in the islets compared to acinar tissue and other organs. This unique vascular architecture is crucial for maintaining optimal islet cell function [6–8]. However, transplantation disrupts islet vasculature, inducing severe hypoxia within 24 hours and contributing to substantial β-cell loss [9]. Unlike whole pancreas transplantation, where vascular reconnection is immediate, islet grafts rely on delayed angiogenesis and vasculogenesis [9–11]. Therefore, strategies aimed at preserving islets viability and promoting revascularization are critical for enhancing islet survival, function and improving transplantation efficacy.

Efforts to prevent hypoxia-induced β-cell loss have included the targeted delivery of lipids, ATP via liposomes and anti-apoptotic agents such as hypoxia-inducible factor-1α (HIF-1α) [12, 13]. While these interventions reduce the adverse effects of oxygen deprivation on islet viability, they fail to restore critical β-cell functions, including glucose responsiveness and glucose-stimulated insulin secretion (GSIS) [14]. To preserve islet functionality while minimizing β-cell death, reoxygenation of isolated islets is essential. Oxygen-carrying biomaterials, such as peroxide-based synthetic polymers, simultaneously mitigate hypoxia and support islet survival within a single microenvironment [15, 16]. However, as hypoxia is a key trigger of proangiogenic signaling through HIF pathways, oxygen supplementation may inadvertently suppress this response, delaying host-mediated revascularization and compromising graft integration [16, 17]. Co-delivery of angiogenic factors, particularly vascular endothelial growth factor (VEGF)—a central mediator of angiogenesis—offers a strategy to overcome this limitation. Enhancing graft revascularization via controlled VEGF release has shown promise in preclinical models [18–20]. Although gene delivery approaches, including viral vectors or transient mRNA-based strategies, can prolong VEGF expression, they are limited by safety concerns such as insertional mutagenesis, immune activation and aberrant vessel formation [21]. More recently, extracellular vesicles (EVs) have attracted attention as alternative pro-angiogenic delivery vehicles, yet their clinical translation is limited by intrinsic heterogeneity in size and cargo composition and an incomplete understanding of their pharmacokinetics and biodistribution [22]. These limitations underscore the need for localized, tunable delivery systems that promote vascular integration while preserving islet viability and function.

**Figure 1:**
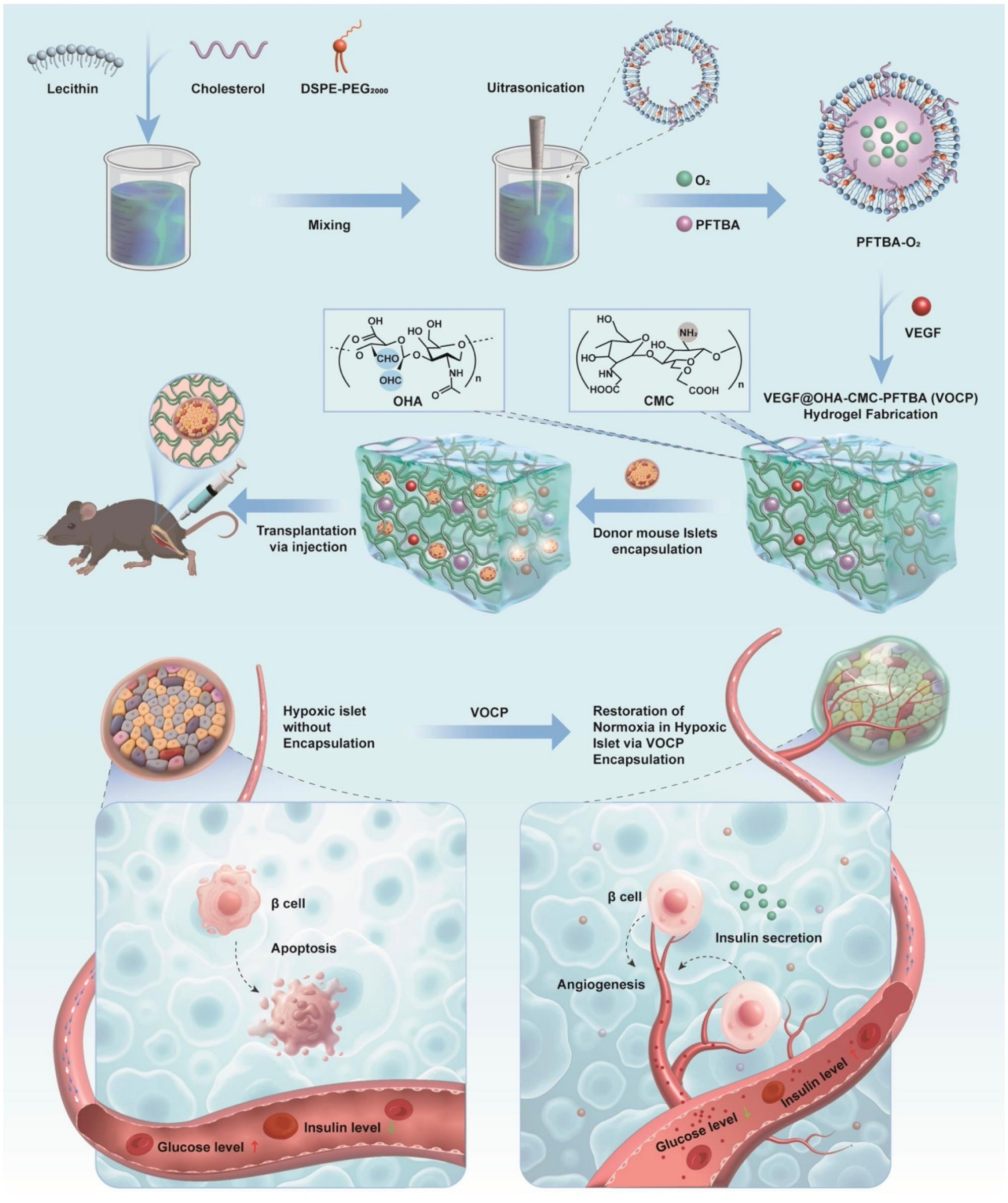
An engineered hydrogel enhances islet graft function and ameliorates hyperglycemia in diabetic mice. Schematic illustration of the design, fabrication and therapeutic application of the dual-functional VEGF@OHA-CMC-PFTBA (VOCP) hydrogel for islet transplantation. Oxygen-loaded PFTBA nanoemulsions (PFTBA-O_2_) were prepared by self-assembly of lecithin and cholesterol to form a lipid shell encapsulating dissolved oxygen, with DSPE-PEG incorporated to improve colloidal stability and biocompatibility. PFTBA-O_2_ nanoemulsions and vascular endothelial growth factor (VEGF) were subsequently embedded within a polysaccharide hydrogel matrix formed via dynamic Schiff-base reactions between aldehyde groups on oxidized hyaluronic acid (OHA) and amino groups on carboxymethyl chitosan (CMC). The resulting injectable VOCP hydrogel enables the sustained release of oxygen and VEGF. Upon encapsulation of freshly isolated mouse islets, the VOCP hydrogel was applied for intramuscular transplantation in streptozotocin (STZ)-induced diabetic mice. By establishing an oxygen-enriched and pro-angiogenic microenvironment, the hydrogel supports islet survival, preserves β-cell function, enhances insulin secretion and ultimately improves glycemic control in diabetic recipients.

Hydrogel-based delivery platforms have become established as versatile vehicles for localized therapy, leveraging their intrinsic biocompatibility, high water content and tunable mechanical properties that closely mimic the extracellular matrix (ECM) [23–25]. These properties make them particularly well suited for islet encapsulation and transplantation. In this study, we engineered a dual-functional injectable hydrogel system composed of oxidized hyaluronic acid (OHA) and carboxymethyl chitosan (CMC), designed to co-deliver oxygen and proangiogenic signals for islet graft support. Leveraging the high oxygen-carrying capacity of perfluorotributylamine (PFTBA) and the proangiogenic activity of VEGF, we constructed a robust OHA–CMC hydrogel network incorporating PFTBA-loaded CMC (CMC–PFTBA) and VEGF-encapsulated OHA (VEGF@OHA), crosslinked via Schiff base chemistry to yield the VEGF@OHA–CMC–PFTBA (VOCP) hydrogel [26]. This system demonstrated favorable injectability, self-healing capacity, tunable degradation and mechanical stability. *In vitro* analysis confirmed that VOCP supports β cell and islet viability, alleviates hypoxia and promotes angiogenesis. *In vivo*, transplantation of VOCP-encapsulated islets into severely diabetic mice resulted in durable glycemic control, highlighting the potential of this platform to improve islet engraftment and function through synergistic oxygenation and vascular support (Figure 1).

## Materials and methods

### Materials

CMC was sourced from Shanghai Yuanye Biotechnology Co., Ltd. (China), with supplier-reported specifications that include a carboxylation degree of 60–100%, a viscosity of 10–80 mPa·s in a 1% aqueous solution at 20°C and a pH range of 6–8 in a 1% aqueous solution. It also contains ≤5 ppm heavy metals (as Pb) and exhibits ≤15% loss on drying. Structural analysis confirmed exclusive substitution at the C6-OH position, with a degree of substitution (DS) of 80% and a degree of deacetylation (DD) of 90%. The material appears as a white to light yellow solid. Lecithin, cholesterol, hyaluronic acid and sodium periodate were purchased from Shanghai McLean Biochemical Co., Ltd. Ethylene glycol was obtained from Shanghai Aladdin Biochemical Technology Co., Ltd., and methoxy PEG phospholipids were provided by Shanghai Pengshuo Biotechnology Co., Ltd. PFTBA was sourced from Sigma-Aldrich (Shanghai) Trading Co., Ltd. All other reagents were obtained from Sigma-Aldrich unless stated otherwise.

### Synthesis of OHA

OHA was synthesized using a previously documented process with minor modifications [26]. In summary, 1.0 g of HA, with a molecular weight of 128.866 kDa as determined by Gel Permeation Chromatography (GPC) as shown in Figure S1A and B, was dissolved in 110 mL of double-distilled water and 0.530 g of NaIO_4_ was dissolved in 20 mL of double-distilled water, thereafter added dropwise to the HA solution at room temperature. The reaction mixture was agitated at ambient temperature in the absence of light for 6 hours to guarantee complete reaction. Subsequently, 1.0 mL of ethylene glycol was used to neutralize the unreacted NaIO_4_, and the mixture was permitted to react for an additional 2 hours at room temperature. The resultant product underwent extensive dialysis (MWCO 12 000) against double-distilled water for 3 days to eliminate residual periodate and ethylene glycol. Following lyophilization, a white, cotton-like substance was acquired.

### Synthesis of oxygen carrier nanoemulsion PFTBA

For the preparation of the PFTBA nanoemulsion, 60.586 mg of lecithin, 10.05 mg of cholesterol and 10.635 mg of DSPE-PEG2000 were solubilized in 10 mL of dichloromethane at room temperature. Following the elimination of dichloromethane using rotary evaporation at 30°C and 156 rpm, the resultant mixture was diluted in 5.0 mL of deionized water and underwent ultrasonic treatment twice (300 W, 3 minutes each) in an ice bath, with a 1-minute delay between treatments. Subsequently, 4.775 mL of the ultrasonically treated solution was amalgamated with 0.225 mL of PFTBA and underwent analogous ultrasonic treatment to produce a PFTBA nanoemulsion. The produced emulsion was subsequently positioned in a hyperbaric oxygen chamber for 30 minutes at room temperature to attain complete oxygen saturation. The solution was ultimately passed through a sterile filter to yield a sterile PFTBA-O_2_ emulsion.

### Formation of hydrogels and functionalized hydrogel (VOCPO_2_)

OHA was reconstituted in phosphate-buffered saline (PBS, 10 mM, pH 7.4) at final concentrations of 2% (w/v), 3% (w/v) and 4% (w/v). CMC was dissolved in the PFTBA-O_2_ emulsion at final concentrations of 1% (w/v), 2% (w/v), 3% (w/v) and 4% (w/v). Equal amounts of the OHA solution and CMC-PFTBA-O_2_ (CPO_2_) emulsion were meticulously combined at room temperature to create OHA-CMC-PFTBA-O_2_ (OCPO_2_) hydrogels. The resultant hydrogels were designated based on the concentrations of the precursor liquids. A hydrogel formulated with 1% OHA and 2% CMC was labeled as OHA1CMC2. VEGF was incorporated into the hydrogel at a final concentration of 200 ng mL^−1^, an effective concentration for endothelial branching, by first dissolving it in an aqueous OHA solution at room temperature, which was then mixed with the CPO_2_ emulsion to facilitate VOCPO_2_ hydrogel formation [27].

### Morphology characteristics

The morphology of PFTBA-O_2_ was examined using transmission electron microscopy (TEM, Talos L120C G2, Thermo Scientific). The hydrated particle size was determined using a Malvern Zetasizer NanoS-90 dynamic light scattering (DLS) device.

The surface morphologies of OHA-CMC hydrogel (OC) and VEGF@OHA-CMC hydrogel without PFTBA (VOC) hydrogels were analyzed via scanning electron microscopy (SEM). Gold-coated frozen hydrogel slices were examined under SEM for structural investigation. Briefly, cylindrical VOC and VOCP hydrogels (Ø 12 mm × 3 mm) were molded and rapidly plunge-frozen in liquid nitrogen to vitrify the internal aqueous microstructures, thereby preventing ice-crystal artifacts. For sample preparation, cryo-fixed samples were lyophilized at ≤−50°C under high vacuum (10^−2^ mbar) for 48 hours. The freeze-dried cylinders were then cleaved perpendicular to the cylindrical axis using a blade to expose pristine cross-sections. Fractured samples were mounted on aluminum stubs, with the exposed cross-sections oriented orthogonally to the electron beam axis, and secured using conductive carbon tape. A uniform Au/Pd layer was sputter-coated (30 mA, 90 seconds) across the fracture plane under argon plasma to ensure surface conductivity while preserving ultrastructural features. Hydrated particle size was measured using a Malvern Zetasizer NanoS-90 dynamic light scattering (DLS) device.

### Gelation and rheological characteristics

The gelation time of the hydrogels was assessed utilizing the vial inversion method. Precursor solutions were introduced into vials and permitted to gel at 25°C. The mixture’s fluidity was assessed, and the gelation time was noted as the moment when the samples ceased to flow upon vial inversion.

The rheological characteristics of the hydrogels were evaluated utilizing an Anton Paar MCR-302e rotational rheometer. Approximately 300 μL of hydrogel was positioned between 25 mm parallel plates with a 1.00-mm gap, and the perimeter was sealed with silicone oil to inhibit water evaporation. The storage modulus (G′) and loss modulus (G″) were assessed in oscillatory mode at a constant strain of 1%. The viscoelastic characteristics of the hydrogels were assessed throughout a frequency spectrum of 10 Hz to 0.1 Hz in frequency-sweep mode at 37°C. The elastic modulus (G′) and viscous modulus (G″) were graphed versus angular frequency to produce rheological curves. Moreover, consistent shear viscosity measurements range from 0.1 to 100 (1 per seconds) shear rates.

### Fabrication, degradation and mechanical characteristics

The hydrogels were fabricated in cylindrical configurations with a diameter of 12 mm and a height of 3 mm. Subsequently, they were submerged in PBS at 37°C. At designated intervals, surplus PBS was meticulously extracted with a pipette, and the mass of the hydrated gels was recorded. The swelling ratio was determined using the subsequent equation:


$$\mathrm{Swelling}\;\mathrm{ratio}(\%)\;=\frac{\;(\mathrm{Ws}-W0)\;}{W0}\times 100\%$$

W0 represents the initial weight of the gels, while Ws denotes the swollen weight of the gels. The *in vitro* degradation of OC, OHA-CMC supplemented with 3% PFTBA (3%OCP) and 5% OCP (*n* = 3) was assessed by measuring weight loss in PBS over a period of 14 days. The initial weight of the lyophilized samples (W0) was measured prior to immersion in PBS at 37°C for specified intervals (1, 4, 7, 10 and 14 days). At each time point, the samples were extracted from PBS, lyophilized and subsequently re-weighed (Wd). The degradation ratio was determined using the following method:


$$\mathrm{Degradation}\;\mathrm{ratio}\;(\%)\;=\frac{\;(W0-\mathrm{Wd})\;}{W0}\times 100\%$$

The mechanical properties of the hydrogels were evaluated using compression testing with a texture analyzer (TA.XTC-18, Shanghai Bosin, China). Cylindrical samples (12 mm diameter, 3 mm height) were stabilized at room temperature (25°C ± 2) for 10 minutes before testing. Samples were placed on the lower plate of the analyzer, and a compressive force was applied at a constant displacement rate of 0.2 mm/min until the yield point was reached. The compressive modulus for each sample was calculated from the corresponding stress–strain curve. Testing was conducted under controlled conditions of 40–60% humidity.

### Self-healing experiment

We initially fabricated two circular VOCP gels using water-based color to evaluate the self-healing behaviors. Following gelation, each VOCP Gel was bisected, and one semi-circle from the red hydrogel was combined with a semi-circle from the transparent hydrogel to create a new structure. Following a 30-minute self-healing period at room temperature, tensile stress was applied manually by rejoining the samples with tweezers, allowing for observation of the adhesion region. A cylinder-shaped hydrogel was fabricated with a diameter of 12 mm and a height of 3 mm. The object was bisected along the vertical axis and subsequently reassembled. Following a 30-minute self-healing period at room temperature, the compression experiment was performed as previously described. An Anton Paar MCR-302e rotational rheometer was utilized to alternately scan VOC and VOCP across three cycles of small strain (1%) and large strain (500%) to investigate their self-healing properties.

### Hydrophilicity assessment of hydrogels

Lyophilized hydrogel samples were processed into thin films for surface wettability analysis. Static water contact angles were measured at room temperature (25 ± 2°C) using a contact angle goniometer. A 4-μL droplet of ultrapure water was gently placed onto the surface of each sample, and the contact angle was recorded to quantitatively assess surface hydrophilicity.

### The blood compatibility experiment of hydrogel

The hemolytic test was used to determine the hemocompatibility of the hydrogel. Erythrocytes were isolated by centrifugation at 4000 rpm for 5 minutes at room temperature, followed by three washes with PBS, also at 4000 rpm for 5 minutes each. Erythrocytes were re-suspended in PBS at a concentration of 2% v/v. Subsequently, 10 mg of lyophilized hydrogel was incubated with 1 mL of erythrocyte suspension at 37°C for a duration of 4 hours. The hydrogel was extracted, and the erythrocyte suspension underwent centrifugation at 4000 rpm for 5 minutes at room temperature. The absorbance of the centrifuged supernatant was recorded at 540 nm. Double distilled water served as the positive control, while PBS functioned as the negative control. The hemolysis ratio was determined using the subsequent formula:


$$\mathrm{Hemolysis}\;\mathrm{ratio}\;(\%)\;=\frac{\;(\mathrm{As}-\mathrm{Ab})\;}{\left(\mathrm{Ap}-\mathrm{Ab}\right)}\times 100\%$$

The absorbance of As refers to the absorbance of the supernatant incubated with hydrogel, while Ab denotes the absorbance of the negative control and Ap indicates the absorbance of the positive control.

### Cell proliferation and cytotoxicity assay

Material extracts, including OC, VOC and VEGF@OHA-CMC hydrogel supplemented with 3% or 5% PFTBA (3% VOCP, 5% VOCP), were prepared according to ISO 10993-12 guidelines by incubating sterilized samples in serum-free DMEM (Thermo) or RPMI-1640 (Life Technology) at 37°C for 24 and 48 hours. Extracts were filter-sterilized (0.22 μm) before use. INS-1 and HeLa cells were plated in 96-well plates and cultured in DMEM or RPMI-1640 medium supplemented with 10% FBS (Sigma) and 1% Penicillin–Streptomycin (Gibco) for 12–24 hours to allow cell adhesion before replacing the medium with 100% extract solutions. After 24–48 hours of incubation at 37°C, cell proliferation and cytotoxicity were assessed using the CCK-8 assay (450 nm). Morphological changes were examined under phase-contrast microscopy, and viability was evaluated per ISO 10993 criteria, with <70% viability indicating cytotoxicity.

### HUVECs tube formation assay

Human Umbilical Vein Endothelial Cells (HUVECs) were plated at a density of 2×10^4^ cells per well in a 48-well plate and resuspended in DMEM Glutamax (Thermo) supplemented with 10% FBS and 1% Penicillin–Streptomycin. Cells were plated on either uncoated plastic surfaces (Control) or substrates pre-coated with 20 μL of LDEV-Free Matrigel (Corning), VOC, 3% VOCPO_2_, 5% VOCPO_2_, 3% VOCP or 5% VOCP hydrogels, which are then carefully placed into each well. Each condition was tested in triplicate.

Following incubation at 37°C for 12 hours, tubule formation was assessed using phase-contrast microscopy at 10× magnification (Nikon). For quantitative analysis, three randomly selected central regions per filter were examined. Images were analyzed using the Angiogenesis Analyzer plugin for Image J, quantifying key parameters including total tubule length, number of nodes and branch points.

### *In vitro* hypoxia model and bulk RNA sequencing in INS-1 cells

INS-1 cells were seeded into transwell inserts placed in 24-well plates at a density of 20 000 cells per well and maintained in RPMI-1640 medium supplemented with 10% fetal bovine serum (FBS) and 1% Penicillin–Streptomycin at 37°C in a humidified incubator (5% CO_2_, 95% air). Upon reaching ∼80% confluence, cells were divided into three groups: normoxia (control), hypoxia and hypoxia treated with 200 μL of VOCPO_2_ hydrogel. Hypoxic exposure was conducted in a hypoxia workstation (0% O_2_, 5% CO_2_, 37°C) for 6 hours. Following treatment, total RNA was extracted using the RNeasy Mini Kit (Qiagen), and RNA quality was assessed using a Bioanalyzer (Thermo Scientific). RNA sequencing was performed by Novogene using the NEBNext Ultra RNA Library Prep Kit for Illumina E7530, followed by sequencing on the Illumina NovaSeq platform to generate 150-bp paired-end reads. Raw reads (FASTQ format) were first processed through in-house perl scripts for quality control, and then aligned to the *Rattus norvegicus* reference genome (mRatBN7.2) using Hisat2 v2.0.5, and gene-level quantification was carried out using featureCounts v1.50-p3.

### Hypoxia-responsed gene set enrichment analysis via GSVA

To quantify hypoxia-related transcriptional activity across experimental groups, gene set variation analysis (GSVA) was performed using normalized expression data from INS-1 cells. A curated hypoxia-responsive gene set comprising 225 genes was assembled from the Molecular Signatures Database (MSigDB). GSVA was applied to compute sample-wise enrichment scores, reflecting the relative activation of hypoxia-associated pathways in each condition. The resulting GSVA scores were used to compare normoxic (Nom), hypoxic (Hyp) and VOCP hydrogel-treated hypoxic (Hyp_VO) groups, followed by statistical comparisons between groups.

### Differential gene expression and pathway enrichment analysis

Differential gene expression analysis was conducted using the DESeq2 R package (1.20.0) to identify genes exhibiting statistically significant changes between experimental groups. Genes with an adjusted *P* values < 0.05 and absolute |log_2_FC| > 0.5 were considered differentially expressed. Results were visualized using volcano plots and hierarchical clustering heatmaps to illustrate expression patterns across groups. To elucidate the biological relevance of the differentially expressed genes, pathway enrichment analysis was performed using the clusterProfiler R package, referencing the Kyoto Encyclopedia of Genes and Genomes (KEGG) database. Enrichment outcomes were visualized using dot plots, bar plots, and pathway network diagrams to facilitate interpretation and support downstream mechanistic insights.

### *In vitro* mouse islet isolation

Mouse islet isolation was performed following a previously established protocol with modifications. Briefly, pancreata were harvested from 8-week-old mice following midline abdominal incision. Cold collagenase P solution (Roche) was injected into the pancreas through the cannulated bile duct to achieve uniform enzymatic perfusion of the tissue. The pancreas was then excised and incubated at 37°C for 16 minutes to facilitate enzymatic digestion. The digested tissue was passed through a mesh screen to remove large undigested fragments, and the filtrate was subjected to a discontinuous dextran gradient (Histopaque, Merck Millipore) for islet purification. Following gradient centrifugation, the solution was allowed to stand for 10–15 minutes to further separate islets from acinar tissue. Purified islets were manually hand-picked and counted under a microscope. All mouse data were obtained from at least three independent islet isolations.

### *In vitro* islets insulin secretion study

Freshly isolated mouse islets were washed three times with PBS, with 20 islets allocated per well. For suspension culture, islets were maintained in RPMI-1640 medium supplemented with 10% FBS and 3% Penicillin–Streptomycin or in the same medium containing 15 μL CMC-3% PFTBA-O_2_ (CPO_2_-S). For encapsulated culture, islets were embedded in 15 μL of hydrogel formulations, including VEGF@OHA-CMC (VOC-E) or VEGF@OHA-CMC-3% PFTBAO_2_ (VOCPO_2_-E), and immersed in culture medium. After 48 or 72 hours of incubation, the medium was removed and islets were washed three times with cold PBS before pre-incubation in 200 μL of 2.8 mM glucose-Krebs buffer (118.5 mM NaCl, 2.54 mM CaCl_2_, 1.19 mM KH_2_PO_4_, 1.19 mM MgSO_4_, 10 mM HEPES, 2% BSA, pH 7.4) at 37°C for 1 hour. Following centrifugation, the supernatant was discarded and replaced with 200 μL of fresh 2.8 mM glucose-Krebs buffer for an additional 1-hour incubation at 37°C to collect basal insulin secretion samples. Sequentially, islets were incubated in 200 μL of 16.8 mM glucose-Krebs buffer for 1 hour to assess glucose-stimulated insulin secretion (GSIS), followed by a final 1-hour incubation in 30 mM KCl-Krebs buffer for further stimulation. Islets were then washed twice with cold PBS and subsequently lysed in RIPA buffer containing protease inhibitors. Lysates were sonicated for 10 seconds for total insulin content and protein quantification. Insulin levels were quantified using mouse insulin ELISA kits (Mercodia).

### *In vitro* islets hypoxia, viability assay

Freshly isolated mouse islets (from 8 to 12-week-old mice) were cultured in RPMI-1640 medium supplemented with 10% FBS and 1% Penicillin–Streptomycin at 37°C in a humidified incubator under 5% CO_2_ and 95% air. Islets were allocated into normoxic and hypoxic groups, with the latter subdivided into four conditions: unencapsulated islets, islets encapsulated in 15 μL VOC, VOCP or VOCPO_2_ hydrogels. Hypoxic cultures were conducted in a hypoxia workstation (0% O_2,_ 5% CO_2,_ 37°C) for 6 hours.

The extent of hypoxia within islets was evaluated using Image-iT™ Hypoxia Reagents (Invitrogen), following the manufacturer’s protocol. Fluorescence imaging was performed using a Nikon fluorescence microscope and a Leica confocal. Hypoxia levels were analyzed in a blinded manner by two independent observers, with fluorescence intensity quantified using Image J. Three independent experiments, each including ∼60 islets from six mice, were analyzed.

Cell viability within islets was evaluated using the Calcein AM/PI Live-Dead Cell Staining Kit (Elabscience) following the manufacturer’s instructions. Calcein-AM, hydrolyzed by intracellular esterases in viable cells, emitted green fluorescence (Ex/Em = 494/517 nm), whereas propidium iodide (PI) selectively entered membrane-compromised cells and bound to double-stranded DNA, producing red fluorescence (Ex/Em = 535/617 nm). Islets were incubated with Calcein-AM and PI for 15 minutes at 37°C, washed with PBS and imaged using a Nikon fluorescence microscope and Leica confocal microscope. The ratio of Calcein-AM to PI signal intensity per islet was quantified. Three independent experiments, each including ∼60 islets from six mice, were analyzed.

### Biosafety *in vivo*

Biosafety evaluation was performed in male C57BL/6 mice (9-week-old, 21–23 g) via subcutaneous implantation of the VOCP hydrogel. Hematological assessments were conducted at baseline (Day 0, PBS-injected sham control), Day 1 and Day 7 post-implantation using a fully automated hematology analyzer (Mindray, BC-30 Vet). Blood samples were analyzed for a comprehensive panel of indices to evaluate systemic compatibility, grouped as follows: (a) Thrombocyte indices: mean platelet volume (MPV, fl), platelet distribution width (PDW, %) and platelet-large cell ratio (P-LCR, %); (b) Leukocyte indices: granulocyte count (Gra#, ×10^9^ L^−1^), lymphocyte count (Lym#, ×10^9^ L^−1^), granulocyte percentage (GRAN, %), lymphocyte percentage (Lym, %), monocyte percentage (Mon, %) and total white blood cell count (WBC, ×10^9^ L^−1^); (c) Erythrocyte indices: hematocrit (HCT, %), hemoglobin concentration (HGB, g dL^−1^), mean corpuscular hemoglobin (MCH, pg), mean corpuscular hemoglobin concentration (MCHC, g dL^−1^), mean corpuscular volume (MCV, fl), red blood cell count (RBC, ×10^12^ L^−1^), red cell distribution width–coefficient of variation (RDW-CV, %) and standard deviation (RDW-SD, fl). These values were compared to sham controls to assess systemic biocompatibility. At each time point (Days 0, 1 and 7), animals were euthanized and major organs—including the heart, lung, liver, kidney, pancreas and spleen—were harvested for histopathological analysis via hematoxylin and eosin (H&E) staining.

### Transplantation and *in vivo* assay

Male C57BL/6 mice (8-week-old, weighing 21-23 g) were used for islet transplantation. Diabetes was induced in mice through a single intraperitoneal injection of streptozotocin (STZ, 150 mg kg^−1^; Sigma-Aldrich), targeting the selective ablation of endogenous β-cells. For intramuscular islet transplantation, 270 isolated islets (approximately 1–2 million cells) were resuspended in 50 μL of VOCPO_2_ hydrogel and transplanted into the leg muscle using a 21-gauge needle. Blood glucose levels were monitored every 2–3 days in the fed state. An intraperitoneal glucose tolerance test (IPGTT) was conducted on Day 6 post-transplantation following an overnight fast. Mice received an intraperitoneal injection of D-glucose (2 g kg^−1^), and blood glucose levels were measured at baseline (fasting, 0 minute) and at 15, 30, 60 and 120 minutes after glucose administration. For glucose-stimulated insulin secretion (GSIS), blood was collected in heparin-coated tubes at fasting state and 30 minutes after glucose administration. Plasma was obtained by centrifugation (2,000*g*, 15 minutes, 4°C), and insulin concentrations were quantified using a mouse insulin ELISA kit (Mercodia). Mice were housed in the Guangzhou Laboratory under standard conditions. All animal experiments were performed in accordance with the National Research Council’s Guide for the Care and Use of Laboratory Animals, and in accordance with protocols approved by the Guangzhou Laboratory’s Animal Care and Use Committee (GZLAB-AUCP-2023-01-A07).

### *In vivo* immunostaining

Whole muscle tissue was harvested from VOCPO_2_ -encapsulated and unencapsulated islet transplant recipients 4 weeks post-transplantation. Tissues were fixed in 4% paraformaldehyde (PFA), cryoprotected and sectioned into 10 µm frozen sections. Sections were permeabilized with 0.1% Triton X-100 for 10 minutes, followed by blocking in 2% donkey serum for 1 hour at room temperature. Primary antibodies, including anti-Insulin (DAKO, #A0564) and anti-CD31 (BD Biosciences, #550274), were applied at 4°C overnight. After PBS washes, sections were incubated with Goat anti-Guinea Pig Alexa Fluor™ 488 and Goat anti-Rat IgG (H + L) Alexa Fluor™ 555 (Invitrogen, # A-21434) for 1 hour at room temperature. Following three PBS washes, sections were mounted with Fluoromount-G containing DAPI (Southern Biotech, #0100-20) and imaged using an Olympus 3000 confocal microscope.

### Statistical analysis

Statistical analyses were performed using GraphPad Prism 9 (GraphPad Software, Inc.). Group comparisons were conducted using one-way/two-way ANOVA followed by Tukey’s multiple-comparison test. Data are expressed as mean ± standard deviation (SD) or mean ± standard error of the mean (SEM). Statistical significance was set at *P* < 0.05, with significance levels denoted as follows: *P < 0.05, **P < 0.01, ***P < 0.001, ****P < 0.0001.

## Results

### VOCP hydrogel formation and optimization

The design and synthesis of VEGF@OHA-CMC-PFTBA (VOCP) hydrogel are illustrated in Figure 1. VOCP consists of a backbone formed by OHA and CMC, incorporating VEGF into OHA (VEGF@OHA) and oxygen carrier nanoemulsion PFTBA into CMC (CMC-PFTBA) to complete the hydrogel structure.

To prepare OHA, hyaluronic acid (HA) was subjected to a ring-opening reaction with sodium periodate, introducing dialdehyde groups into the dimer units of HA (Figure S1C). The oxidation of HA to OHA was validated using multiple spectroscopic techniques. In the ^1^H NMR spectra, OHA displayed two novel peaks at 4.8 ppm and 4.9 ppm, corresponding to hemiacetal protons and vicinal hydroxyl groups, absent in unmodified HA (Figure S1D). Additionally, Fourier transform infrared (FT-IR) spectroscopy identified a characteristic aldehyde peak at 1728 cm^−1^ (-C = O- stretching), further confirming successful oxidation (Figure S1E). The degree of oxidation was quantified as 48.22% via hydroxylamine hydrochloride titration, consistent with previous findings (Figure S1F).

**Figure 2:**
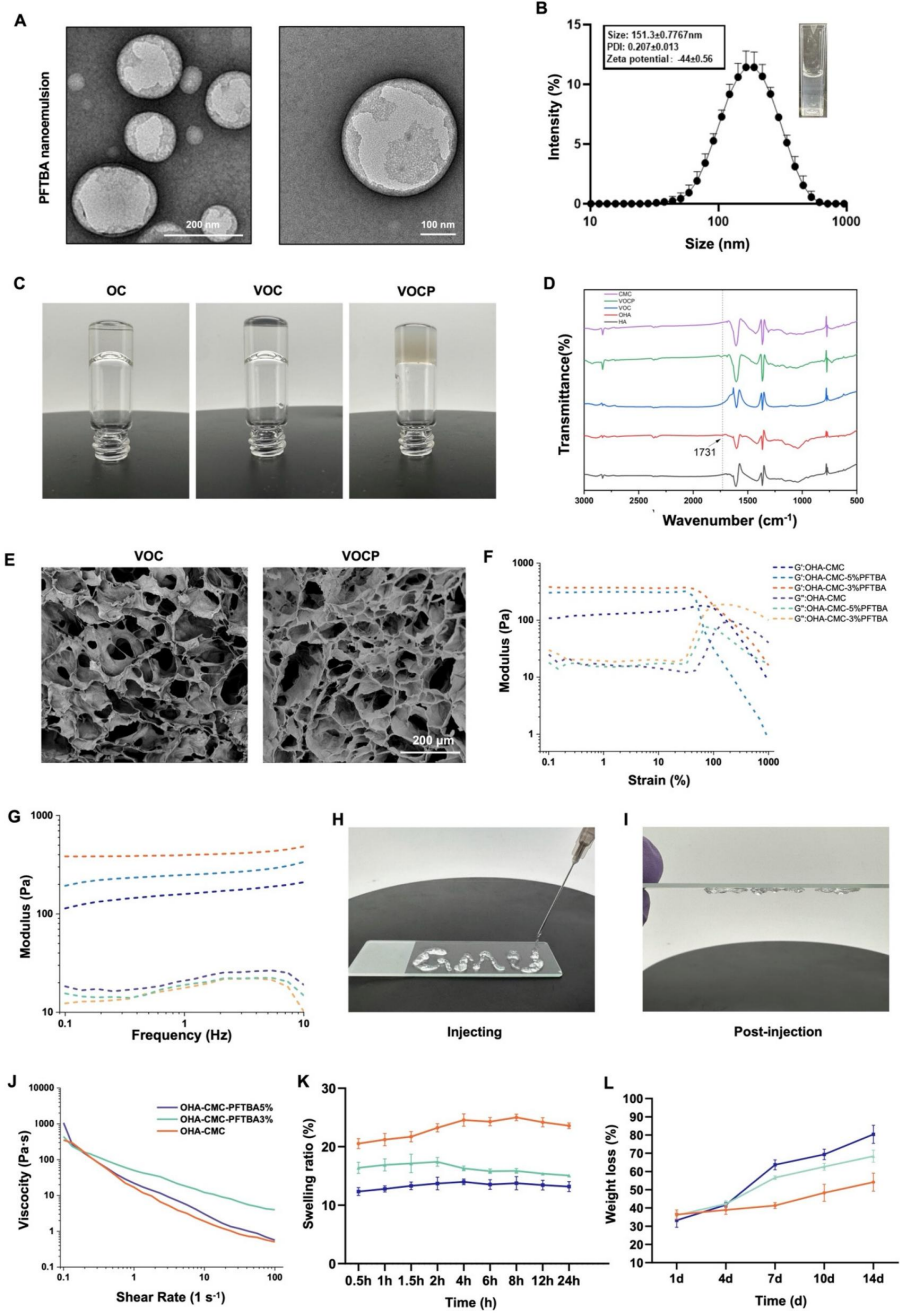
Fabrication and characterization of VOCP hydrogels. (**A**) TEM images of PFTBA nanoemulsions. Scale bars: 200 nm (left) and 100 nm (right). (**B**) Size distribution and zeta potential of PFTBA nanoemulsions measured by dynamic light scattering (DLS). Inset: Translucent appearance of the oxygen-loaded PFTBA solution. (**C**) Digital images presenting the gelation process of OHA-CMC (OC), VEGF@OHA-CMC (VOC) and VOCP hydrogels. (**D**) Fourier transform infrared (FT-IR) spectra of HA, OHA, CMC and the representative hydrogels VOC and VOCP, confirming the formation of a Schiff base (-C=N-) bond between OHA and CMC, as indicated by the characteristic peak at 1733 cm^−1^ (arrow), highlighting the covalent linkage within the hydrogel network. (**E**) Cross-sectional SEM images of VOC and VOCP scaffolds showing interconnected porous structures. Scale bar: 200 μm. (**F**) Strain amplitude sweep (γ = 0.1%–1000%) assessing the mechanical stability of the hydrogels under varying strain. (**G**) Frequency sweep behavior (ω = 0.1–10 Hz) of hydrogels, indicating dynamic viscoelastic properties. (**H and I**) Injectability assessment demonstrating that VOCP hydrogel can be extruded through a standard medical syringe and recover its solid-like elasticity post-injection. (**J**) Shear viscosity profiles of OC, 3% VOCP, and 5% VOCP hydrogels, exhibiting pronounced shear-thinning behavior. (**K**) Degradation rate of hydrogels quantified by weight loss indicated by percentage (%) over time (d: days). (**L**) Swelling properties of hydrogels, measured as the swelling ratio (%) over time (h: hours), evaluating water absorption capacity.

To enable oxygen delivery to hypoxic islets, a PFTBA nanoemulsion was synthesized. The emulsion consisted of a PFTBA core encased in a lipid monolayer composed of lecithin, cholesterol and distearoyl phosphoethanolamine-polyethylene glycol 2000 (DSPE-PEG2000), subsequently subjected to oxygen loading. Transmission electron microscopy (TEM) validated the development of spherical and flexible nanoemulsion structures (Figure 2A). Dynamic light scattering (DLS) analysis indicated a consistent hydrodynamic diameter of 151.3 nm, accompanied by a polydispersity index of 0.207 (Figure 2B). The translucent appearance of the oxygen-loaded PFTBA solution (Figure 2B, inset) confirmed the successful synthesis.

OHA-CMC (OC), VOC and VOCP Hydrogels were formed via a Schiff base reaction between aldehyde groups on OHA and amino groups on CMC (Figure 2C). This reaction was validated by the presence of a characteristic peak at 1733 cm^−1^ in the FT-IR spectra, corresponding to -C = O- bonds within the hydrogel structure (Figure 2D). Leveraging this reaction, the VOC hydrogel was synthesized by combining VEGF-loaded OHA (VEGF@OHA) with CMC, while the VOCP hydrogel incorporated both VEGF@OHA and PFTBA-loaded CMC (CMC-PFTBA) as an oxygen carrier (Figure 2C). To optimize gelation conditions, OHA polymers with different degrees of oxidation were synthesized, and precursor solutions with varying concentrations of OHA, CMC and PFTBA were formulated. The gelation time was evaluated using a vial inversion method (Figure S1G), demonstrating that OHA2CMC2 formulations reliably formed hydrogels within 120 seconds, regardless of the PFTBA concentrations used. The findings indicate that OHA2CMC2 is the most suitable formulation for future research. The duration of 120 seconds is sufficient for an operator with brief training to encapsulate islets and transplant them into the graft region. Extended gelation time may result in the displacement of the transplant from its intended location.

### Robust mechanical properties enable injectability of VOCP hydrogel with controlled degradation

VOCP was designed as an injectable and degradable delivery vehicle. To ensure its suitability for *in vivo* applications, its physical properties, including microstructure, mechanical properties, degradation rate and swelling ratio must be systematically assessed. To evaluate the influence of PFTBA concentration on the hydrogel’s physical characteristics, OC-based hydrogels containing 3% and 5% PFTBA were prepared for comparative analysis.

Scanning electron microscopy (SEM) presented the porous microarchitecture of the hydrogels, with pore sizes exceeding 60 μm (Figure 2E). This structure supports the infiltration of vascular endothelial cells and facilitates nutrient exchange and metabolic waste removal, critical for biological functionality.

Rheological testing demonstrated that the hydrogels transitioned from a solid-like state (storage modulus, G′ > loss modulus, G″) to a liquid-like state (G″ > G′) under an oscillatory strain of approximately 100% (Figure 2F), indicating shear-thinning behavior suitable for injectability. Frequency sweep analysis showed stable G′ and G″ values across a range of 0.1–10 Hz (Figure 2G), confirming the hydrogel’s structural stability under dynamic conditions. Compression tests further revealed that all hydrogels exhibited resistance to compressive strain. Stress increased linearly with strain up to 60%, and the network remained intact, maintaining shape integrity up to 40% strain (Figure 2F). This property facilitates smooth extrusion from syringes, rendering the hydrogels highly injectable, as demonstrated in Figure 2H and I. All hydrogel formulations exhibited shear-thinning behavior, evidenced by a decrease in viscosity with increasing shear rates (Figure 2J).

Degradation studies indicated that the hydrogels exhibited slow and controlled mass loss, retaining approximately 30% of their initial weight after 14 days of incubation (Figure 2K). This degradation rate aligns with the requirements for *in vivo* applications such as inducing angiogenesis. Swelling studies showed that the hydrogels could absorb over 10 times their dry weight in water, reflecting their ability to facilitate nutrient migration (Figure 2L).

Overall, incorporating PFTBA into the hydrogel scaffold significantly enhanced its modulus and viscosity, resulting in improved mechanical properties and structural stability. The VOCP hydrogel also exhibited controlled degradation, high swelling capacity and excellent injectability, making it an ideal candidate for islet transplantation applications.

### VOCP exhibits self-healing properties and excellent hydrophilic ability

The hydrogel network is susceptible to disruption by external forces, such as bending and compression, particularly under strong mechanical challenges following transplantation. Hydrogels with self-healing properties can restore their structural integrity after rupture, enhancing their durability and functional lifespan. VOCP hydrogels exhibit notable flexibility, enabling resistance to bending and compression especially the 3% PFTBA (Figure S3). This resilience is attributed to the flexible polymer chains of OHA/CMCS and the dynamically bonded hydrogel network, which collectively confer mechanical adaptability and robustness.

**Figure 3:**
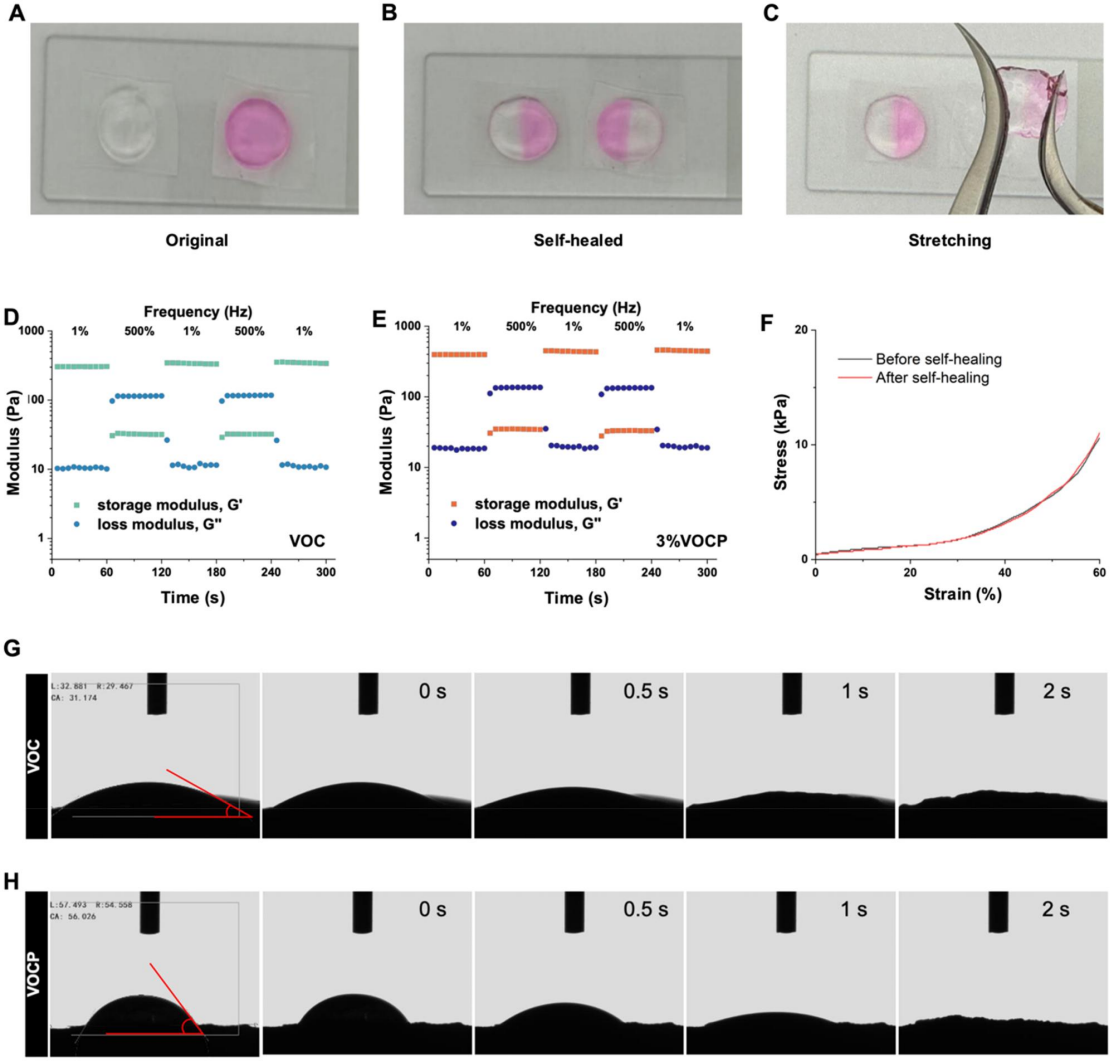
VOCP demonstrates self-healing property and hydrophilicity. (**A–C**) Self-healing capability demonstrated by cutting the hydrogel into halves and rejoining them, resulting in the formation of healed interfaces. (**D and E**) Alternating step-strain sweep experiments for VOC (**D**) and 3% VOCP (**E**), applying strain cycles of 1% and 500% with 60-second intervals. (**F**) Stress-strain curves of the self-healing hydrogels, illustrating their mechanical resilience. (**G and H**) Contact angle measurements revealing the hydrophilic properties of the hydrogels. Water droplet spreading on the surface yielded a contact angle of 31.2° for the VOC hydrogel (**G**) and 56.0° for the VOCP hydrogel (**H**), indicating strong hydrophilicity in both formulations.

To evaluate the self-healing ability of VOCP, two circular hydrogels of different colors were prepared, cut into halves and recombined by joining halves from each circle. Following 30 minutes of self-healing at room temperature, the halves merged seamlessly to create a complete circle, as indicated by the uniform color at the edges (Figure 3A and B). The self-healed hydrogel’s robustness was validated by suspending one end without any separation of the fused sections (Figure 3C). A rheological recovery experiment further quantified the self-healing capacity. Upon strain amplitude sweep assay, the data revealed the critical strain point at approximately 100%, where the storage modulus (G′) intersected with the loss modulus (G″). Beyond this threshold, the hydrogel network was disrupted, as indicated by a sharp decline in G′. When subjected to 500% strain, the G′ of VOCP dropped from 396 Pa to 35 Pa but recovered to its original value upon returning the strain to 1% (Figure 3D and E). This repeated fracture and recovery cycle confirmed the rapid and efficient self-healing behavior of VOCP. Compressive testing further demonstrated VOCP’s resilience. Stress–strain curves and the maximum strain and stress values remained consistent after self-healing, confirming its mechanical robustness (Figure 3F). The resilience is due to the flexible polymer chains of OHA/CMC and the dynamically bonded hydrogel network, which together provide mechanical adaptability and robustness.

To assess the surface wettability of VOC and VOCP hydrogels, contact angle measurements were performed. Upon deposition of a water droplet, the contact angles measured 31.2° for VOC and 56.0° for VOCP, demonstrating favorable hydrophilic properties in both hydrogel formulations (Figure 3G and H). The low contact angles reflect effective water spreading, indicative of strong surface hydrophilicity essential for nutrient diffusion and cell interaction.

### VOCP is a bioactive hydrogel with pro-angiogenic property and blood compatibility

To evaluate the potential cytotoxic effects or impact on cell proliferation of the newly synthesized VOCP hydrogel, HeLa (human cell line) and INS-1 (rat cell line) cells were cultured under the following conditions: without hydrogel (Control), with OHA-CMC hydrogel (OC), with VEGF@OHA-CMC hydrogel without PFTBA (VOC), or with VEGF@OHA-CMC hydrogel supplemented with 3% or 5% PFTBA (3%VOCP, 5% VOCP). Cell viability was assessed at 24 and 48 hours using a colorimetric assay to monitor proliferation and potential cytotoxicity.

**Figure 4:**
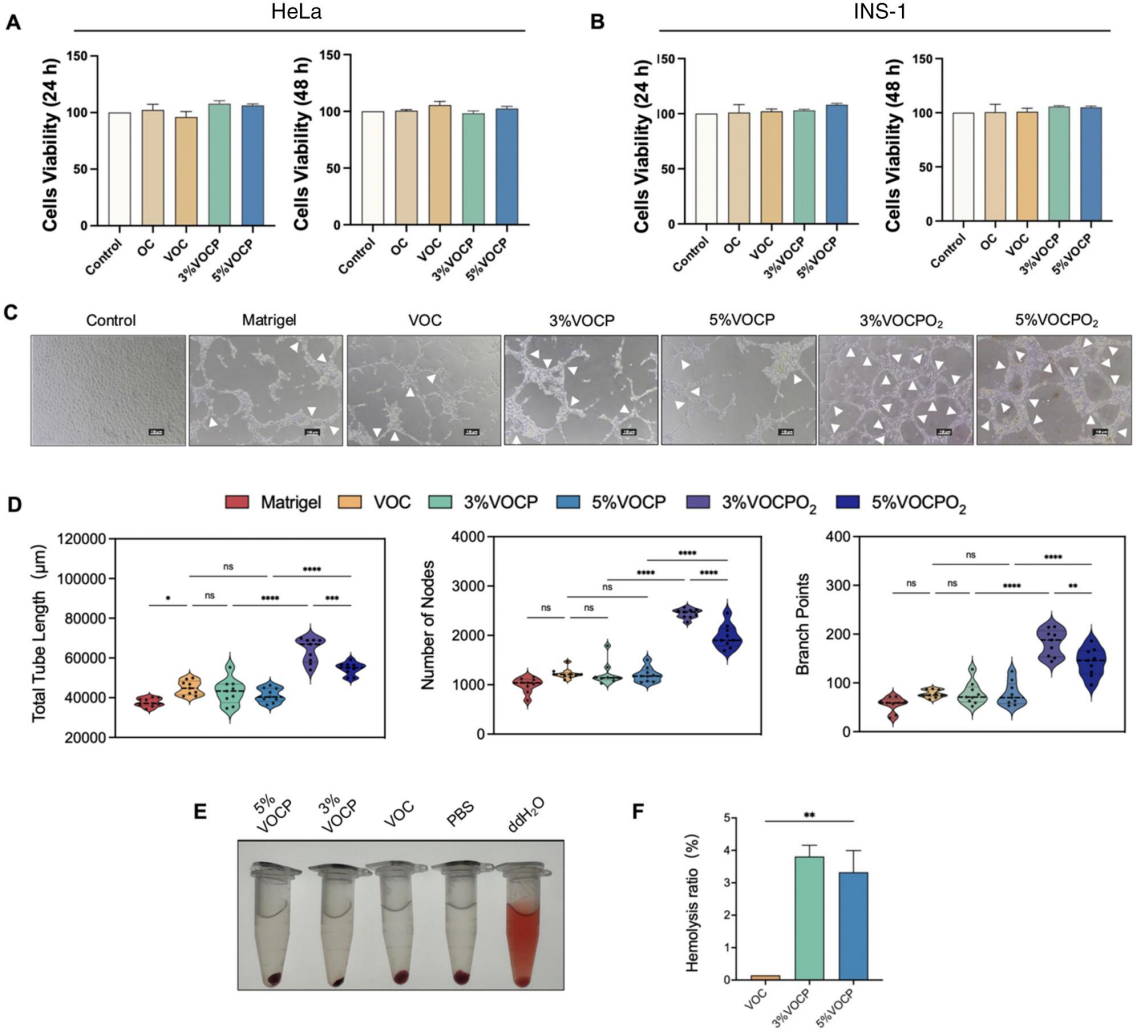
VOCPO_2_ demonstrates biocompatibility and angiogenic potential *in vitro*. (**A and B**) Cell viability of HeLa (**A**) and INS-1 (**B**) cells measured using a colorimetric assay after 24 hours and 48 hours of culture under various conditions: OHA-CMC (OC), VEGF@OHA-CMC (VOC), VEGF@OHA-CMC-3% PFTBA (3% VOCP) and VEGF@OHA-CMC-5% PFTBA (5% VOCP) (*n*=3). Statistical analysis was conducted using one-way ANOVA, with data presented as mean ± SD, showing no significant difference relative to the control group. (**C**) Brightfield images of HUVEC tube formation in control conditions (no gel) and on gels treated with Matrigel, VOC, 3% VOCP, 5% VOCP, 3% VOCPO_2_ and 5% VOCPO_2_. Scale bar: 100 µm. White arrow: Branch points. (**D**) Quantification of tube length, number of nodes and branch points from the HUVEC tube formation assay after 24 hours of culture (*n* = 3; three images per batch, three batches in total). (**E and F**) Hemolytic activity of different materials in blood. Hemolysis ratio of VOC and 3%VOCP and 5%VOCP. Statistical analysis was conducted using one-way ANOVA followed by Tukey’s multiple comparisons test. Data are presented as mean ± SD, with significance levels indicated as *P < 0.05, **P < 0.01, ***P < 0.001 and ****P < 0.0001.

For both HeLa and INS-1 cells, cell viability in the OC-, VOC- and VOCP-treated groups was comparable to the control group after 24 hours of incubation. No significant differences were observed between the 3% VOCP and 5% VOCP groups. Viability remained consistent after an additional 24 hours under all conditions, indicating that neither the components of VOCP nor the hydrogel itself exerted any cytotoxic effects on human or rat cells *in vitro* (Figure 4A and B).

Angiogenesis *in vitro* relies on the ability of endothelial cells to organize into tubule-like structures when cultured on an appropriate extracellular matrix [28]. Matrigel—a matrix derived from murine tumors has become the standard substrate for tubule formation assays [29]. To assess the pro-angiogenic potential of VEGF-incorporated OHA-CMC hydrogels as a potential alternative to Matrigel, human umbilical vein endothelial cells (HUVECs) were cultured overnight on uncoated plastic dishes (Control) or substrates coated with Matrigel, VOC, 3% VOCP, 5% VOCP, 3% VOCPO_2_, or 5% VOCPO_2_ and cultured overnight (Figure 4C and D). The primary distinction between VOCP and VOCPO_2_ lies in the presence or absence of oxygen-loaded PFTBA. Robust tubule formation was observed across all hydrogel-coated conditions (Figure 4C). Quantitative analysis demonstrated that VOCPO_2_-coated substrates significantly enhanced angiogenesis, as indicated by increased tube length, node formation and branch points compared to the control or VOCP groups (Figure 4D). Notably, 3% VOCPO_2_ exhibited the most pronounced pro-angiogenic effect, surpassing 5% VOCPO_2_ (Figure 4D). These findings highlight the importance of incorporating an optimal oxygen-delivery capacity into VOCP for effective angiogenesis. The 3% VOCPO_2_ was adopted for the following functional evaluation.

The biosafety of VOCP was assessed through hemolysis analysis. While the water control group exhibited complete hemolysis, hydrogel-PBS solutions (10% v/v) demonstrated negligible hemolysis ratios (below 5%), indicating excellent blood compatibility of VOCP (Figure 4E and F).

### VOCPO_2_ attenuates hypoxia-induced gene expression programs

To evaluate whether oxygen supplementation via the VOCP hydrogel could mitigate hypoxia-induced transcriptional responses, INS-1 cells were cultured under normoxic (Nom, 100% O_2_) or hypoxic (Hyp, 0% O_2_) conditions. The hypoxic group was further divided into two subgroups: untreated hypoxia (Hyp) and VOCP hydrogel-treated hypoxia (Hyp_VO).

**Figure 5:**
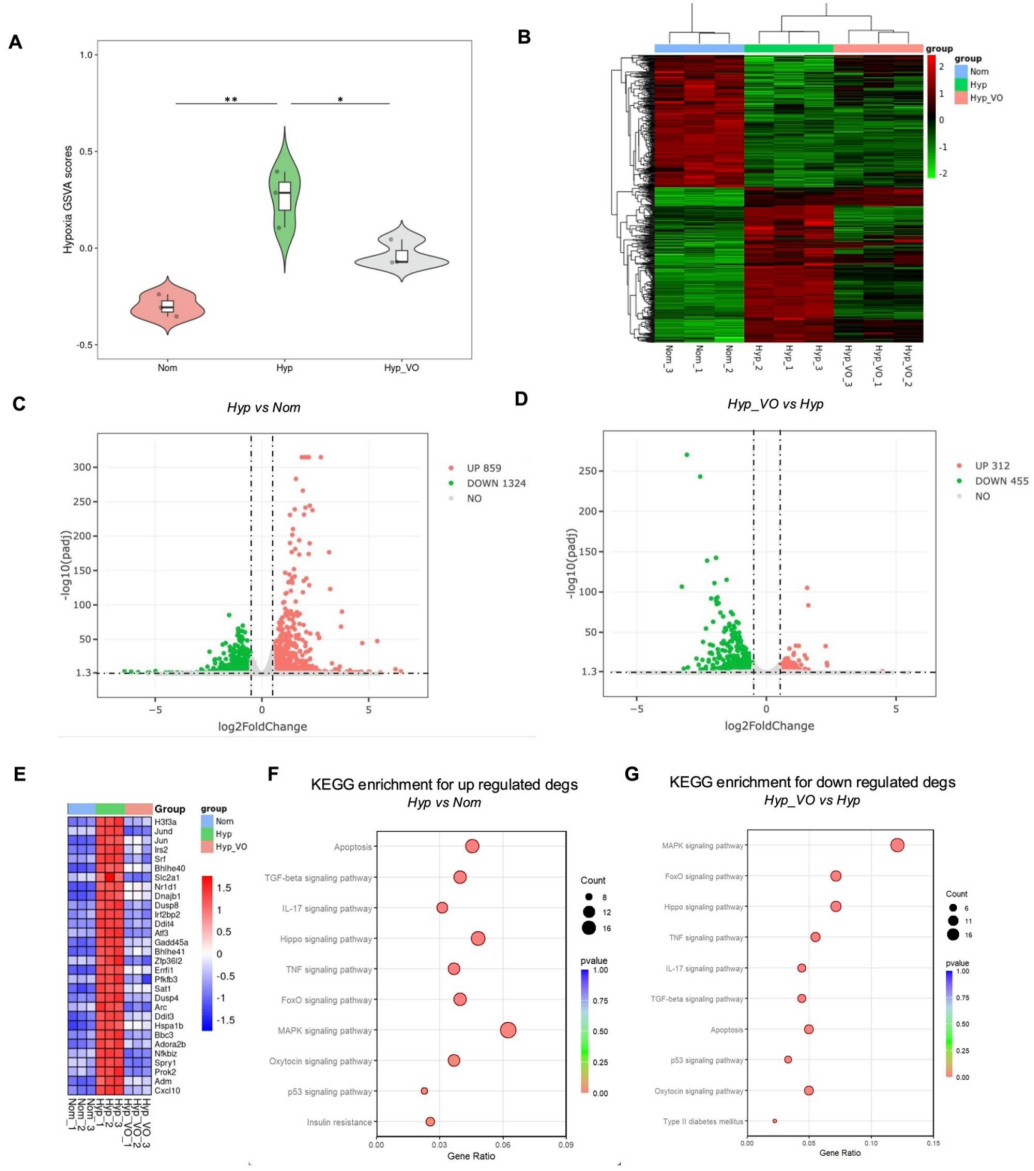
VOCPO_2_ attenuates hypoxia-induced gene activity in INS-1 cells. (**A**) Gene Set Variation Analysis (GSVA) was performed using a curated hypoxia-related gene set (200 genes, including 25 hypoxia-specific markers from MSigDB) to assess hypoxia-associated transcriptional activity across Normal (Nom), hypoxia without hydrogel (Hyp) and hypoxia with VOCPO_2_ hydrogel supplementation (Hyp_VO) groups. VOCPO_2_ treatment significantly reduced hypoxia-related GSVA scores (*n* = 3). Statistical comparisons between groups were conducted using unpaired two-tailed *t*-tests. Data are presented as mean ± SD; *P < 0.05, **P < 0.01 versus the Hyp group. (**B**) Hierarchical clustering analysis of differentially expressed genes across Nom, Hyp and Hyp_VO groups, with a cutoff of |log_2_FC| > 0.5 and adjusted *P* < 0.05. (**C**) Volcano plot showing differential gene expression between Hyp and Nom groups, identifying 859 upregulated and 1324 downregulated genes. (**D**) Volcano plot of gene expression differences between Hyp_VO and Hyp groups, with 312 genes upregulated and 455 downregulated. (**E**) Expression profile of selected hypoxia-related genes across all conditions. (**F and G**) KEGG pathway enrichment analyses of upregulated genes (Hyp vs. Nom) and downregulated genes (Hyp_VO vs. Hyp). Pathways upregulated under hypoxia included FoxO signaling, insulin resistance, oxytocin, apoptosis, TNF and IL-17 signaling—all associated with diabetes and cell death progression. Notably, these pathways were significantly attenuated upon VOCPO_2_ supplementation.

Gene Set Variation Analysis (GSVA) was performed using a curated hypoxia-related gene set consisting of 225 genes from the MSigDB database. GSVA scores revealed a marked elevation of hypoxia-associated gene activity in the Hyp group, which was significantly reduced upon VOCP treatment (Hyp_VO), approaching levels observed under normoxia (Figure 5A).

Differential gene expression analysis between Hyp and Nom conditions identified 859 upregulated and 1,324 downregulated genes, many of which are key mediators of hypoxic stress responses (Figure 5B and C). Notably, in the Hyp_VO group, expression of these hypoxia-induced genes was substantially suppressed, confirming the oxygen-delivering capability of the VOCP hydrogel (Figure 5D and E).

KEGG pathway enrichment analysis of upregulated genes in the Hyp group highlighted the activation of FoxO, insulin resistance, oxytocin, apoptosis, TNF and IL-17 signaling pathways, all of which are implicated in diabetes progression and hypoxia-induced apoptosis. These pathways were significantly downregulated in the Hyp_VO group, indicating that VOCPO_2_ hydrogel supplementation effectively blunts hypoxia-driven molecular responses (Figure 5F and G).

### VOCPO_2_ preserves islet function by mitigating apoptosis during *in vitro* culture

**Figure 6:**
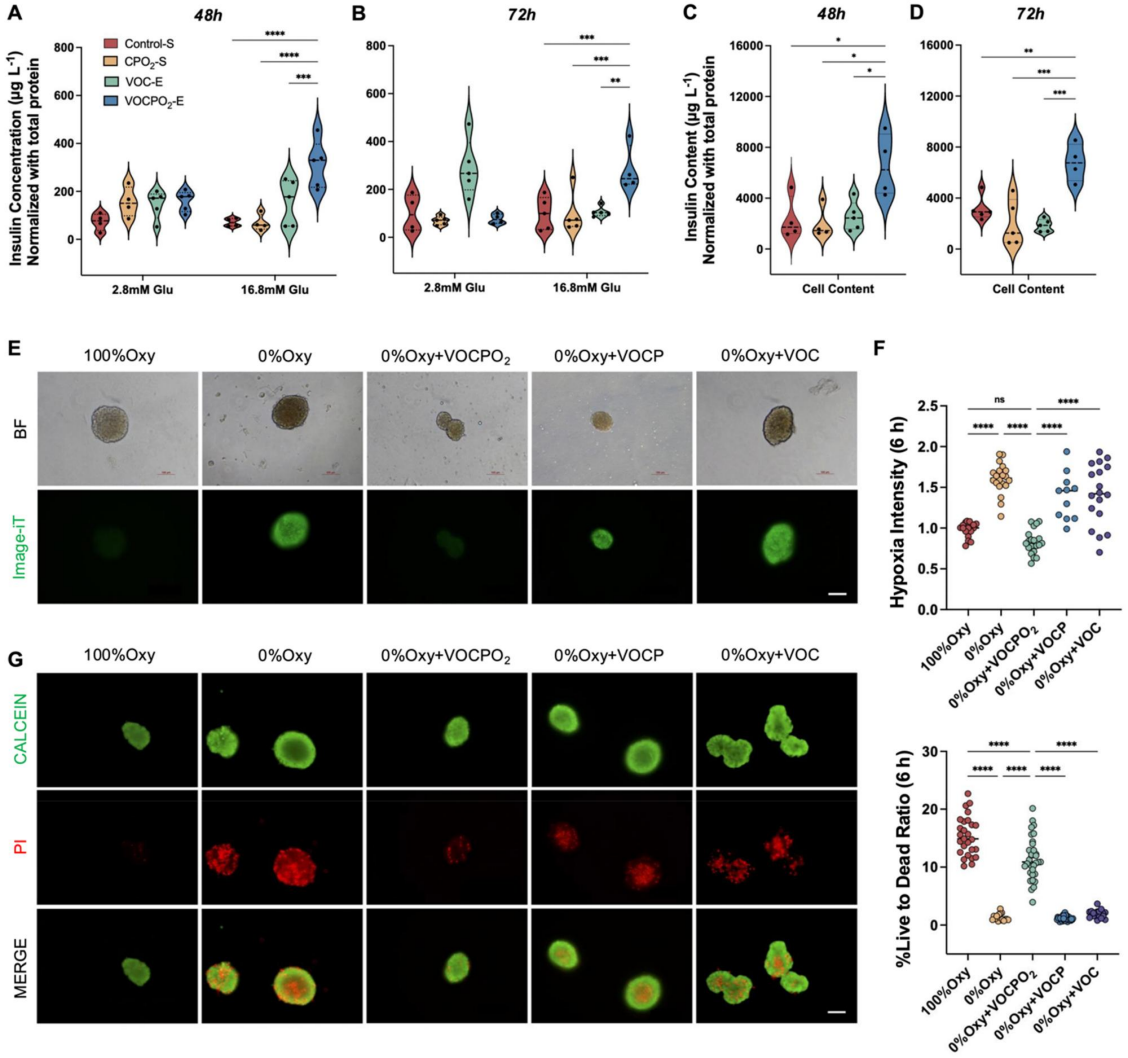
VOCPO_2_ enhances insulin secretion in mouse islets by mitigating intra-islet hypoxia and reducing cell death. (**A–D**) Functional assessment of encapsulated mouse islets *in vitro*. Glucose-stimulated insulin secretion (GSIS) was assessed in mouse islets cultured under various conditions: islets suspended in culture media (Control-S) or CMC-PFTBAO_2_ (CPO_2_-S) and islets encapsulated in hydrogel VEGF+OHA+CMC (VOC-E) or VEGF@OHA+CMC+PFTBAO_2_ (VOCPO_2_-E) after 48 hours (**A**) and 72 hours. (**B**). Insulin secretion was measured from media collected under low glucose (2.8 mM) and high glucose (16.8 mM) conditions. Total insulin content was quantified from cell lysates after 48 hours (**C**) and 72 hours (**D**) of culture under these conditions, followed by GSIS assessment. Insulin concentrations were determined via ELISA, normalized to total protein content, and expressed as µg L^−1^. (**E and F**) Hypoxia assessment in mouse islets under different oxygen conditions. (**E**) Bright field and fluorescent images of mouse islets cultured under normoxia (100% O_2_) or hypoxia (0% O_2_) for 6 hours. Hypoxia groups included unencapsulated islets and islets encapsulated with VOCPO_2_, VOCP or VOC. Hypoxia levels were evaluated using the fluorogenic compound Image-iT, which fluoresces in response to decreased oxygen concentrations. Scale bar: 100 µm. (**F**) Quantification of hypoxia levels from panel (**E**) based on fluorescence intensity (*n*=3, 4-7 data points per batch). (**G and H**) Apoptosis measurement in mouse islets under different oxygen conditions. (**G**) Live/dead cell fluorescence imaging of mouse islets cultured under varying oxygen conditions for 6 hours. Live cells were visualized with calcein (CALCEIN) green, and dead cells with propidium iodide (PI) red. Scale bar: 100 µm. (**H**) Quantification of cell viability from panel (**G**) based on fluorescence intensity (*n* = 3, 6–12 data points per batch). Statistical analyses were performed using one-way or two-way ANOVA. Data are presented as mean ± SD with **P < 0.01, ***P < 0.001, ****P < 0.0001 relative to 100% Oxy group.

To examine the effect of an oxygen-enriched environment on islet functionality *in vitro*, freshly isolated mouse islets were cultured under various conditions and assessed for insulin secretion in response to glucose stimulation using the glucose-stimulated insulin secretion (GSIS) assay (Figure 6A–D).

The first experiment aimed to evaluate the influence of gel encapsulation on insulin secretion, comparing islets cultured in standard suspension to those encapsulated in gel matrices for 48 hours (Figure 6A) and 72 hours (Figure 6B). In suspension cultures, islets were maintained in either standard media (Control-S) or media supplemented with CMC-3% PFTBAO_2_ (CPO_2_-S) to assess the effect of oxygen enrichment alone, without gel support, on islet function. Insulin secretion levels were similar between the Control-S and CPO_2_-S groups, indicating that oxygen supplementation alone does not significantly affect islet functionality. In contrast, islets encapsulated in oxygen-enriched hydrogel, VEGF@OHA-CMC-3% PFTBAO_2_ (VOCPO_2_-E), showed a 4-fold increase in insulin secretion at 16.8 mM glucose after 48 hours (****P < 0.0001) and a 2.8-fold increase after 72 hours (***P = 0.0007) compared to Control-S. Additionally, while islets encapsulated in hydrogel without oxygen supplementation, VEGF@OHA-CMC (VOC-E), failed to enhance insulin secretion under 16.8 mM glucose stimulation, VOCPO_2_-E induced significantly higher insulin secretion than both CPO_2_-S (48 h: ****P < 0.0001; 72 h: ***P = 0.0007) and VOC-E (48 h: ***P = 0.0007; 72 h: **P = 0.0011) (Figure 6A and B). Following 16.8 mM glucose stimulation, 30 mM KCl was introduced, but no significant differences in insulin secretion were observed among the groups under KCl stimulation (Figure S4A and B). After completing the GSIS assay, islets were lysed to assess intracellular insulin content. The VOCPO_2_-E group exhibited higher intracellular insulin levels compared to Control-S, CPO_2_-E and VOC-E (Figure 6C and D). These findings suggest that VOCPO_2_ enhances both secreted and intracellular insulin levels *in vitro*.

Intra-islet hypoxia can lead to islet dysfunction. To further investigate whether VOCPO_2_, in combination with an oxygen-supplemented gel matrix, mitigates hypoxia, islets were cultured under normoxic (100% Oxy) or hypoxic (0% Oxy) conditions for 6 hours in suspension culture. Additionally, islets encapsulated with VEGF@OHA+CMC + 3% PFTBAO_2_ (VOCPO_2_), VEGF@OHA+CMC + 3% PFTBA (VOCP) or VEGF@OHA+CMC (VOC) were cultured under hypoxic conditions. Hypoxia levels were assessed using the fluorogenic compound Image-iT, which fluoresces under decreased oxygen concentrations (Figure 6E; Figure S4C). Bright-field and fluorescent imaging (Figure 6E) and confocal microscopy (Figure S4C) were used to monitor intracellular hypoxia intensity. Islets cultured in suspension under hypoxic conditions exhibited a 63% increase in hypoxia intensity compared to those cultured under normoxia (****P < 0.0001). Encapsulation with VOCPO_2_ significantly reduced hypoxia intensity by 48% under hypoxic conditions, bringing it to a level comparable to normoxic islets (*P *= 0.2440 vs. 100% Oxy), and indicating a protective effect against hypoxia (****P < 0.0001 vs. 0% Oxy). In contrast, islets encapsulated with VOCP, which includes 3% PFTBA but lacks oxygen supplementation, failed to alleviate hypoxia to the same extent as VOCPO_2_ (****P < 0.0001: 0% Oxy + VOCPO_2_ vs. 0% Oxy + VOCP). Similarly, islets encapsulated with VOC displayed hypoxia intensity comparable to VOCP under hypoxic conditions (Figure 6E and F; Figure S4C). These findings highlight the importance of oxygen supplementation in the gel matrix for mitigating intra-islet hypoxia.

Oxygen deprivation induces apoptosis in pancreatic islet cells [11]. To evaluate cell viability under hypoxic conditions, mouse islets were cultured for 6 hours under various conditions, including normoxia (100% Oxy), hypoxia (0% Oxy) and hypoxia with VOCPO_2_, VOCP or VOC encapsulation. Live cells were stained with calcein green, dead cells with propidium iodide (PI) red, and visualized using fluorescence (Figure 6G) and confocal microscopy (Figure S4D). Cell viability was quantified as the live-to-dead cell ratio. Islets cultured under 0% Oxy exhibited significantly increased cell death, particularly within the islet core, compared to those cultured under 100% Oxy (Figure 6G; Figure S4D). The live/dead ratio decreased sharply from 15.360 ± 3.357 (100% Oxy) to 1.330 ± 0.596 (0% Oxy, ****P < 0.0001) (Figure 6H). However, VOCPO_2_ encapsulation under hypoxia (0% Oxy + VOCPO_2_) significantly mitigated hypoxia-induced apoptosis, restoring the live/dead ratio to 11.460 ± 3.583 (****P < 0.0001). In contrast, islets encapsulated with VOCP or VOC failed to prevent hypoxia-induced apoptosis, as indicated by significantly lower live/dead ratios compared to 0% Oxy + VOCPO_2_ (****P < 0.0001) (Figure 6H). These results highlight the efficacy of VOCPO_2_ in preserving islet cell viability under oxygen-deprived conditions.

### VOCP demonstrates favorable biocompatibility and hemocompatibility with no detectable bioaccumulation *in vivo*

**Figure 7:**
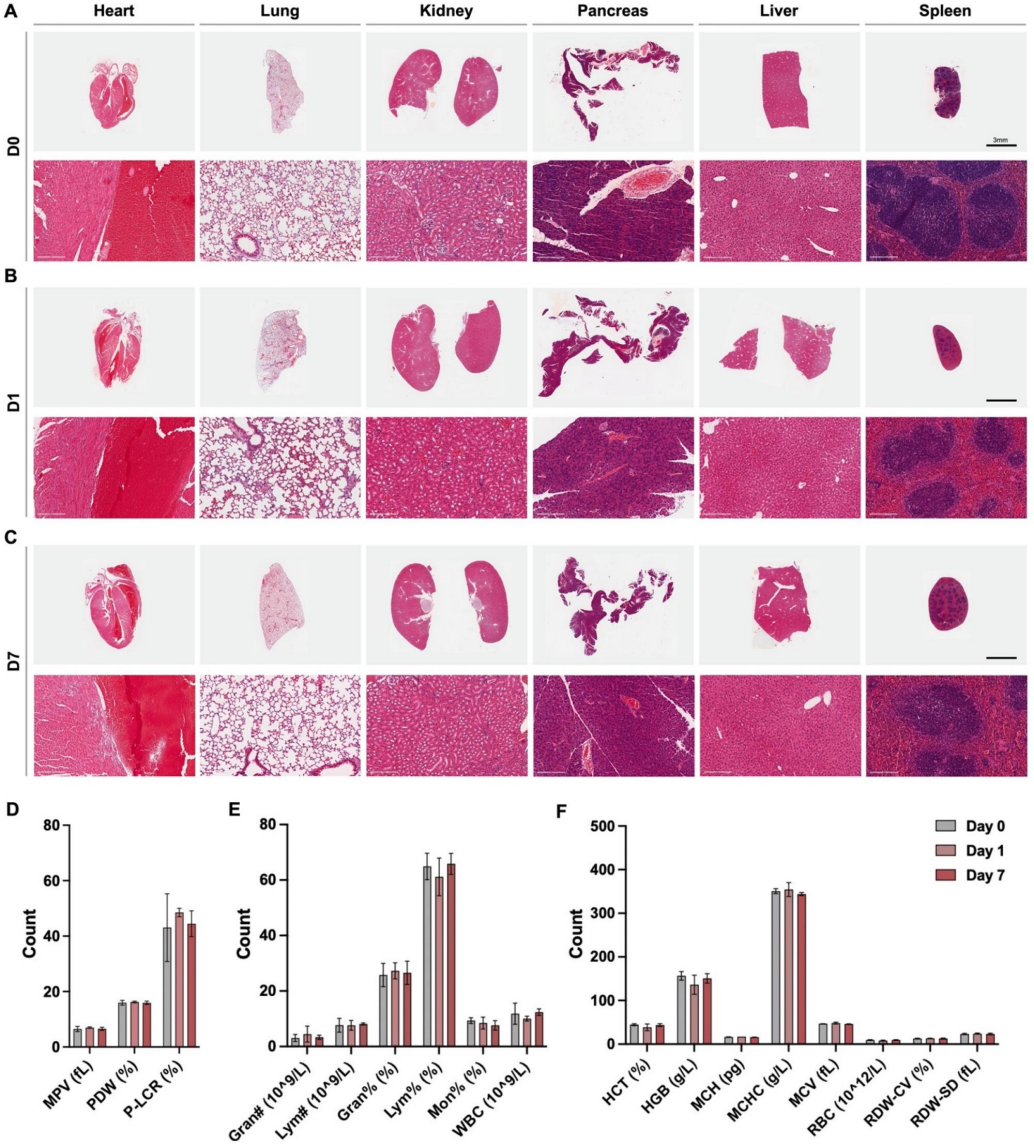
Systemic inflammatory analysis: Comprehensive biocompatibility and hemocompatibility profiling of VOCPO_2_ hydrogel. (**A–C**) Pathomorphological analysis: Representative H&E-stained sections of major organs (heart, lung, kidney, pancreas, liver and spleen) collected prior to implantation (**A**, Day 0), and at 1 day (**B**, Day 1) and 7 days (**C**, Day 7) post-implantation. Scale bars: top panels, 3 mm; bottom panels, 200 µm. (**D–F**) Hemocompatibility assessment of the VOCP hydrogel through whole blood analysis at Day 0, Day 1 and Day 7 post-implantation. Parameters include (**D**) thrombocyte, (**E**) leukocyte and (**F**) erythrocyte indices. Thrombocyte indices: mean platelet volume (MPV, fl), platelet distribution width (PDW, %) and platelet-large cell ratio (P-LCR, %). Leukocyte indices: granulocyte count (Gra#, ×10^9^ L^−1^), lymphocyte count (Lym#, ×10^9^ L^−1^), granulocyte percentage (GRAN, %), lymphocyte percentage (Lym, %), monocyte percentage (Mon, %) and total white blood cell count (WBC, ×10^9^ L^−1^). Erythrocyte indices: hematocrit (HCT, %), hemoglobin concentration (HGB, g dL^−1^), mean corpuscular hemoglobin (MCH, pg), mean corpuscular hemoglobin concentration (MCHC, g dL^−1^), mean corpuscular volume (MCV, fl), red blood cell count (RBC, ×10^12^ L^−1^), red cell distribution width–coefficient of variation (RDW-CV, %) and red cell distribution width–standard deviation (RDW-SD, fl). Data presented as mean ± SD (*n*=3).

To evaluate the biosafety and tissue compatibility of the VOCP hydrogel, we conducted comprehensive histological and hematological assessments following subcutaneous implantation. Major organs—including the heart, lungs, kidneys, pancreas, liver and spleen—were harvested at baseline (Day 0, PBS-injected sham), 1 day post-implantation (acute phase) and 7 days post-implantation (chronic phase). H&E staining revealed no evidence of inflammatory infiltration, structural damage or pathological abnormalities across all time points, suggesting the absence of systemic toxicity or bioaccumulation of degradation byproducts (Figure 7A–C). These findings support the favorable biocompatibility profile of VOCP *in vivo*.

To further evaluate systemic biocompatibility, we performed complete blood count (CBC) analyses following subcutaneous implantation of VOCP. Hematological profiles were assessed at Day 1 and Day 7 post-implantation and compared to PBS-injected controls (Day 0). No statistically significant differences were observed across thrombocyte indices (MPV, PDW, P-LCR) (Figure 7D), leukocyte indices (Gra#, Lym#, GRAN, Lym, Mon, WBC) (Figure 7E) or erythrocyte indices (HCT, HGB, MCH, MCHC, MCV, RBC, RDW-CV, RDW-SD) (Figure 7F). These results confirm that VOCP implantation does not elicit hematological abnormalities, further supporting its systemic safety *in vivo*.

### VOCPO_2_ serves as a functional scaffold for islet transplantation, preserving islet functionality *in vivo*

**Figure 8:**
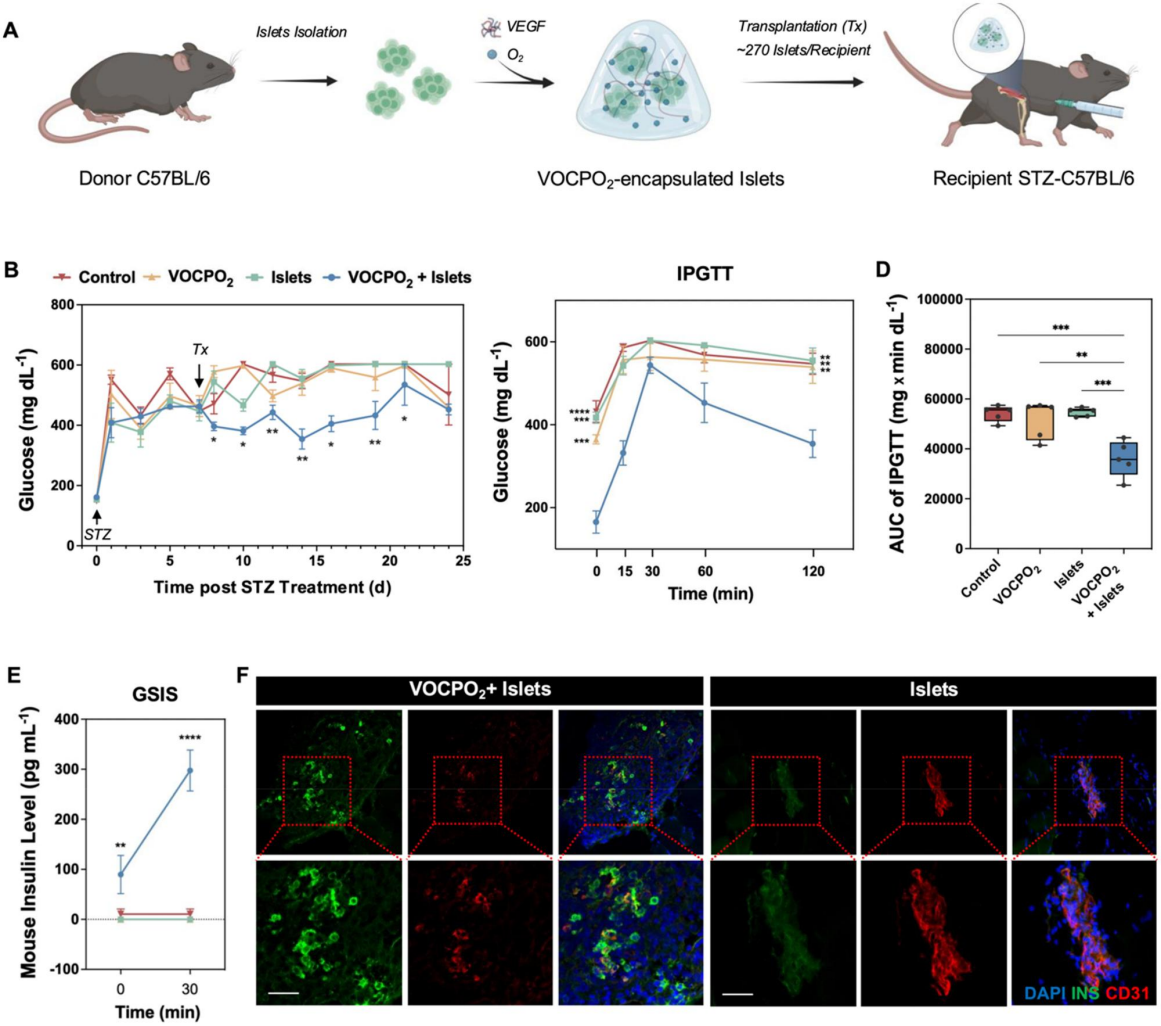
VOCPO_2_-encapsulated mouse islets mitigate hyperglycemia in STZ-treated C57BL/6J mice. (**A**) Schematic representation of mouse islet isolation, VOCPO_2_ encapsulation and intramuscular transplantation (Tx) into STZ-treated recipient C57BL/6J mice. (**B**) Non-fasting blood glucose levels (mg dL^−1^) in mice transplanted with no graft (Control), VOCPO_2_ alone, islets alone, or VOCPO_2_-encapsulated islets (VOCPO_2_+Islets). Approximately 270 islets were transplanted per mouse in the Islets and VOCPO_2_+Islets groups. Day 0 marks STZ treatment initiation, and transplantation occurred 7 days post-STZ treatment (*n*=5 per group). (**C**) Glycemic control assessed by intraperitoneal glucose tolerance test (IPGTT) after 1-week post-transplantation. (**D**) Area under the curve (AUC) of the IPGTT glucose profile (mg X min dL^−1^). (**E**) Blood insulin response to glucose stimulation, measured by glucose-stimulated insulin secretion (GSIS) and expressed as insulin concentration (pg mL^−1^). (**F**) Representative images of islet grafts from VOCPO_2_-encapsulated islets and unencapsulated islets transplant recipients, stained for insulin (INS) and CD31. Scale bar: 25 µm. Statistical analysis was conducted using one-way ANOVA or two-way ANOVA, with data presented as mean ± SEM. Statistical significance is indicated as follows: *P < 0.05, **P < 0.01, ***P < 0.001, ****P < 0.0001.

Transplantation of pancreatic islets from organ donors can repair physiological blood glucose regulation in diabetic patients [30]. To investigate whether VOCPO_2_ can serve as a functional scaffold for islet transplantation *in vivo*, freshly isolated donor mouse islets were encapsulated with VOCPO_2_ (VOCPO_2_+islets) or left unencapsulated (islets) and transplanted into streptozotocin (STZ)-treated diabetic recipient mice (Figure 8A). As negative controls, equivalent volumes of VOCPO_2_ gel alone (VOCPO_2_) or PBS (Control) were injected into recipient mice. Blood glucose levels were monitored from the onset of STZ treatment (Day 0) through transplantation (Tx) on Day 7 and up to Day 24 (Figure 8B). Mice receiving VOCPO_2_+islets exhibited a significant reduction in hyperglycemia, whereas the other groups showed no improvement, confirming that the islets encapsulated within the VOCPO_2_ gel were responsible for restoring glucose homeostasis (Figure 8B).

To assess the *in vivo* functionality of the implants, an intraperitoneal glucose tolerance test (IPGTT) was conducted 6 days post-transplantation. Mice grafted with VOCPO_2_+islets exhibited significantly lower fasting blood glucose levels (165.2 ± 26.88 mg dL^−1^) compared to the islets-only group (*P *= 0.0002), the VOCP group (*P *= 0.0008) and the control group (*P *< 0.0001) (Figure 8C). Glycemic control was further evaluated by calculating the area under the curve (AUC) for glucose levels during the IPGTT. Mice implanted with VOCPO_2_+islets demonstrated the lowest AUC among all groups, showing a significant reduction compared to islets-only, VOCP, or control groups (Figure 8D). These results indicate that VOCPO_2_+islets provide superior glycemic regulation.

To confirm that the transplanted VOCPO_2_-encapsulated islets were directly responsible for glucose regulation, GSIS test was performed (Figure 8E). Following 30 minutes of glucose stimulation, the VOCPO_2_+ islet grafts exhibited a significant increase in insulin secretion, demonstrating their viability and functional capacity. In contrast, insulin production was negligible in other engrafted mice, reaffirming the successful depletion of pancreatic β-cells by STZ induction and attributing the observed metabolic control to the functionality of the VOCPO_2_+ islet grafts (Figure 8E).

To confirm that insulin secretion was mediated by functional β-cells preserved within VOCPO_2_-encapsulated islets *in vivo*, grafts transplanted into muscle were retrieved from both VOCPO_2_-encapsulated and unencapsulated islet transplant recipients. Immunostaining revealed the presence of insulin-positive (INS^+^) cells within VOCPO_2_-encapsulated islets, with a well-integrated intra-graft vascular network labeled by CD31. In contrast, islets were scarcely detectable within the muscle tissue of unencapsulated transplant recipients (Figure 8F). These findings indicate that the VOCPO_2_ hydrogel not only functioned as a structural scaffold to maintain the engraftment site but also provided a supportive microenvironment, enhancing nutrient availability and preserving β-cell survival and function.

Additionally, VOCPO_2_-encapsulated islets effectively maintained body weight and improved survival in severely diabetic mice, preventing fatal outcomes associated with hyperglycemia (Figure S5A and B).

## Discussion

In this study, we developed a dual-functional hydrogel-based encapsulation platform incorporating the oxygen-carrying molecule PFTBA and bioactive VEGF to enhance islet transplantation. This injectable and biodegradable hydrogel was engineered to create an oxygen-enriched, VEGF-supplemented microenvironment, supporting islet survival and function. Systematic characterization demonstrated its excellent cytocompatibility, proangiogenic properties and tunable oxygen-carrying capacity. By facilitating PFTBA-mediated oxygen delivery, the hydrogel effectively restored normoxic conditions, mitigating hypoxia-induced cell death in β cells and primary islets. Furthermore, it enhanced islet function, as evidenced by improved insulin secretion, and significantly ameliorated hyperglycemia in diabetic mice post-transplantation. Addressing key challenges in islet transplantation, this platform (1) mitigates islet hypoxia through controlled oxygen delivery, (2) preserves islet viability and function in both *in vitro* and *in vivo* settings and (3) promotes glycemic regulation, ultimately improving transplantation efficacy in severe diabetes.

Various strategies have been explored for the development of injectable *in situ*-forming hydrogels, ranging from photopolymerization to chemical crosslinking [31, 32]. However, the clinical translation of these strategies remains challenging due to potential cytotoxicity associated with chemical crosslinkers and the risk of prolonged irradiation compromising the integrity of biologically active substances [31]. We demonstrated an injectable self-healing hydrogel created using dynamic Schiff base connections between OHA and CMC. The reversible crosslinking network, established through aldehyde-amine conjugation, eliminates the need for cytotoxic crosslinkers such as glutaraldehyde, commonly used in traditional photo-polymerized or ionically crosslinked systems (e.g. Ca^2+^-alginate) [33]. This dynamic network imparts shear-thinning properties and self-repair capability, allowing the hydrogel to maintain its structural integrity during injection while safeguarding the encapsulated islets from mechanical stress. Precise modulation of the molecular weight and degree of substitution in OHA/CMC enabled the optimization of gelation kinetics (∼120 seconds at 37°C) and degradation profiles, with ∼30% mass retention over 14 days. This tunability ensured a balance between structural integrity (storage modulus G′ = 400 Pa) and controlled degradation, aligning with the timeline of neovascularization to support islet engraftment. The integration of PFTBA nanoemulsions (200 nm diameter, PDI < 0.2) endowed the hydrogel with precise oxygen regulation through two complementary mechanisms: (1) modulation of oxygen diffusion via crosslink density and (2) controlled oxygen release from lipid-soluble PFTBA, governed by Henry’s law [34].

A critical determinant of successful islet transplantation in T1D is the local transplant microenvironment, which poses both immediate and long-term challenges, including early inflammatory responses and sustained hypoxia due to insufficient revascularization [35]. Strategies that modulate this microenvironment, such as embedding growth factors within matrices or coating islets with bioactive materials, have shown promise in overcoming these site-specific barriers. Pancreatic β-cells are particularly sensitive to oxygen deprivation, with hypoxia-induced ER stress being a key contributor to post-transplantation β-cell apoptosis and insulin secretion dysfunction [11]. We observed the formation of a pronounced hypoxic core within islets after just 6 hours of exposure to 0% O_2_, reflecting the acute vulnerability of islet grafts. Remarkably, islets supplemented with VOCPO_2_ hydrogel maintained a normoxic microenvironment even under sustained hypoxia, highlighting its capacity to mitigate oxygen deprivation during the critical engraftment phase. Transcriptomic profiling under hypoxia revealed the upregulation of multiple stress-associated pathways, including FoxO signaling, insulin resistance, oxytocin, apoptosis, TNF and IL-17, all of which are implicated in diabetes progression and hypoxic β-cell dysfunction [36–38]. These signatures were significantly attenuated in the VOCPO_2_-treated group, suggesting a direct effect of oxygen delivery on hypoxia-responsive gene regulation. Correspondingly, VOCPO_2_ significantly reduced core apoptosis and preserved islet viability, outperforming VOC and VOCP formulations lacking the oxygen-carrying component. These effects are attributed to the dissolved oxygen reservoir provided by PFTBA within the hydrogel matrix, which buffers local oxygen tension and thereby alleviates hypoxia-induced cellular stress [35]. By sustaining a more permissive oxygen microenvironment in a non-toxic manner, this oxygenation strategy effectively reduces hypoxia-associated cell death and supports cell survival and function [39]. Functionally, this protection translated into enhanced insulin secretory capacity: VOCPO_2_-treated islets demonstrated superior insulin synthesis and secretion under hypoxic stress. In line with this, diabetic mice receiving VOCPO_2_-encapsulated islet grafts exhibited restored insulin responsiveness to glucose stimulation by Day 6 post-transplantation, coinciding with the previously reported oxygen release duration of PFTBA (up to 144 hours) [40]. Together, these findings establish VOCPO_2_ as an oxygen carrier that supports β-cell survival, maintains insulin secretory function, and effectively alleviates hypoxia-driven graft attrition, offering a clinically relevant strategy to enhance islet transplantation outcomes.

Islet isolation from donors leads to the loss of native capillaries, creating a significant barrier to successful transplantation and therapeutic efficacy. The production of angiogenic factors, particularly VEGF, plays a crucial role in establishing the dense vascularization necessary for islet survival and function [7]. During embryogenesis, interactions between endothelial and endocrine cells, along with the development of functional blood vessels, are thought to guide pancreatic differentiation and morphogenesis [41]. Attempts to enhance the revascularization of transplanted islets face significant challenges due to the lack of a clear understanding of the key ligands, receptors, cells and underlying mechanisms involved. Endothelial cells contributing to graft vascularization originate from recipient vasculature, intra-islet endothelial cells and, to a lesser extent, bone marrow-derived cells [42–45]. While tissue-engineered approaches incorporating endothelial cells have been explored, the risk of immune rejection and teratoma formation cannot be fully eliminated in such complex tissues composed of cells with high proliferative capacity [46, 47]. Beyond cellular contributions, vascular remodeling also relies on extracellular matrix (ECM) components, which support endothelial organization and islet function. Although ECM-derived hydrogels have shown promise in preserving islet viability, their clinical application is limited by complex fabrication processes and donor scarcity [48]. In this context, a VEGF-releasing hydrogel represents a compelling alternative to enhance islet engraftment. The VOCPO_2_ hydrogel, incorporating VEGF at 200 ng mL^−1^, significantly enhanced endothelial tube formation in HUVEC cultures, demonstrating robust angiogenic activity. While oxygen-enriched microenvironments are known to promote neovascularization in other regenerative contexts [49], their role in islet transplantation has remained largely unexplored. Our *in vitro* results show that oxygenation synergizes with VEGF delivery, as evidenced by greater tube formation with VOCPO_2_ compared with VOCP, suggesting enhanced vasculogenic potential. Consistent with this, *in vivo* transplantation of VOCPO_2_-encapsulated islets preserved INS^+^ β-cells and promoted intra-islet capillary neogenesis, indicated by CD31^+^ structures in close proximity to insulin-positive cells. Functionally, VOCPO_2_-treated grafts achieved sustained glycemic control over a 3-week period compared with non-encapsulated controls. Together, these findings indicate that early oxygen supplementation supports islet survival immediately after transplantation, while sustained VEGF release drives vascular remodeling and nutrient exchange, ultimately improving β-cell preservation and long-term graft function in diabetic mice.

The STZ-induced diabetic mouse model employed in this study differs from conventional models, as blood glucose levels in control mice reached approximately 600 mg dL^−1^, exceeding the typical 400 mg dL^−1^ reported in other studies [50]. This was intentionally designed to simulate severe hyperglycemia, providing a more rigorous assessment of therapeutic efficacy. This heightened baseline glucose level may explain why blood glucose in VOCPO_2_-engrafted mice did not fall below 200 mg dL^−1^, the threshold for normoglycemia, despite the hydrogel’s beneficial effects on islet survival and function [51]. Extended *in vivo* monitoring will provide deeper insights into the long-term efficacy and translational potential of this approach for clinical application.

The injectable and biodegradable VOCP hydrogel presents a promising strategy for overcoming key barriers in islet transplantation for T1D by simultaneously improving the oxygen microenvironment and enabling controlled VEGF release. The hydrogel not only supports islet survival by mitigating hypoxia-induced apoptosis, preserving β-cell viability and enhancing islet function, but also amplifies the pro-angiogenic effects of VEGF, promoting vascular network formation. A single dose of VOCPO_2_ provided dual benefits, preserving islet viability and insulin independence while accelerating vascularization in the short term. Furthermore, the controlled release of VEGF further improved islet revascularization, which enhanced metabolic control and ultimately contributed to prolonged recipient survival in STZ-induced diabetic mice. Given its tunable physicochemical properties and capacity to simultaneously regulate oxygenation and therapeutic factor release, this hydrogel platform has applications that extend beyond islet transplantation for T1D. It may also provide therapeutic benefit in type 2 diabetes (T2D), particularly in advanced or late-stage patients with impaired islet function, where sustained metabolic support is critical. In this context, the platform could support both donor-derived and stem cell-derived islets, enhancing graft survival, functional maturation and glycemic regulation in the metabolically compromised T2D microenvironment.

More broadly, this adaptable platform is well suited for organ transplantation and tissue regeneration strategies that require rapid vascular integration and sustained metabolic support. Potential applications include myocardial repair, where epicardial delivery within an oxygen-enriched microenvironment could mitigate post-infarction hypoxia while enabling the controlled co-delivery of pro-angiogenic factors such as HGF or VEGF; infected wound healing, through the combined delivery of oxygen and antimicrobial peptides (e.g. LL-37) to promote epithelialization and infection control, particularly in settings where prolonged or severe hypoxia impairs cell function, collagen deposition and immune defense [52], and neural regeneration, in which spatially regulated oxygen gradients, together with sustained nerve growth factor (NGF) release, may guide axonal growth and support functional recovery.

Despite these encouraging findings, several limitations should be acknowledged. First, the use of a severe STZ-induced diabetic model, characterized by extreme hyperglycemia, may underestimate the therapeutic efficacy of the hydrogel system. Employing a milder STZ regimen or increasing the number of encapsulated islets could potentially improve glycemic rescue, particularly given that a fraction of islets may undergo damage or functional impairment during isolation prior to engraftment. Second, although the current study demonstrates enhanced short-term recovery of glycemic control, extended *in vivo* monitoring will be necessary to assess long-term graft stability, durability of vascularization and sustained metabolic benefit. Such longitudinal evaluation will be essential to fully establish the clinical translational potential of this hydrogel platform.

## Supplementary Material

rbag024_Supplementary_Data

## Data Availability

All data supporting the findings of this study are available within the paper and Supplementary Materials.

## Conclusion

In summary, this study demonstrates the successful development of a dual-functional, injectable, and self-healing hydrogel platform designed to overcome the critical barriers of hypoxia and insufficient vascularization in islet transplantation. By integrating the oxygen carrier PFTBA and the pro-angiogenic factor VEGF within a dynamic OHA/CMC matrix, we created a synergistic microenvironment that significantly enhances graft outcomes. Our findings establish that the VOCPO_2_ hydrogel effectively mitigates acute hypoxia by restoring normoxic conditions during the critical early engraftment phase, which in turn suppresses hypoxia-induced ER stress and apoptotic pathways such as FoxO and TNF signaling. This protection preserves islet function by sustaining high insulin secretory capacity and maintaining β-cell viability, even within the rigorous physiological demands of a severe in vivo diabetic model. Furthermore, the platform accelerates revascularization by promoting robust intra-islet capillary neogenesis through the synchronized delivery of oxygen and VEGF, ensuring the long-term metabolic exchange necessary for survival. While the severity of the STZ-induced diabetic model utilized in this study underscores the potency of this platform, further longitudinal studies are required to evaluate the long-term stability and eventual clinical translation of the graft. Beyond T1D, the tunable nature of this oxygen-enriched delivery system holds broad therapeutic potential for other regenerative medicine applications, including myocardial repair, neural regeneration, and chronic wound healing, where metabolic support and rapid vascular integration are paramount for success.
