# Supplementary material for: A dual-functional injectable polysaccharide hydrogel incorporating oxygen-carrying nanoemulsion and VEGF for enhancing islet survival and glycemic control in diabetic mice
Source: Regen Biomater. 2026 Mar 5;13:rbag024. doi: 10.1093/rb/rbag024 (PMC13175250; doi:10.1093/rb/rbag024)
Supplement: rbag024_Supplementary_Data [file rbag024_supplementary_data.docx]

**A Dual-Functional Injectable Polysaccharide Hydrogel Incorporating Oxygen-Carrying Nanoemulsion and VEGF for Enhancing Islet Survival and Glycemic Control in Diabetic Mice**

Yuwei Zhang^1,4^, Zhaoyu Sun^1,4^, Miao Liu^1^, Jinxin Pang^1^, Shanshan Huang^1^, Hongyang Liu^1^, Huabin Zheng^1^, Jie Li^1^, Yifan Feng^1,3^, Jiang Ouyang^2, 5^,Tao Xu^1,3, 5^ and Wen Du^1, 5^

1 Key Laboratory of Biological Targeting Diagnosis, Therapy and Rehabilitation of Guangdong Higher Education Institutes, the Fifth Affiliated Hospital & School of Biomedical engineering, Guangzhou Medical University, Guangzhou,Guangdong, 511436, China

2 Guangzhou Institute of Cancer Research, the Afiliated Cancer Hospital & School of Biomedical Engineering, Guangzhou Medical University, Guangzhou 510180, China

3 Guangzhou Laboratory, Guangzhou, Guangdong, 510320, China

4 These authors contributed equally

5 Correspondence: email address: [duwen@gzhmu.edu.cn](mailto:duwen@gzhmu.edu.cn)；[xutao@ibp.ac.cn;](mailto:xutao@ibp.ac.cn;) ouyangjiang1989@gmail.com;

**Supplemental Figures**

**
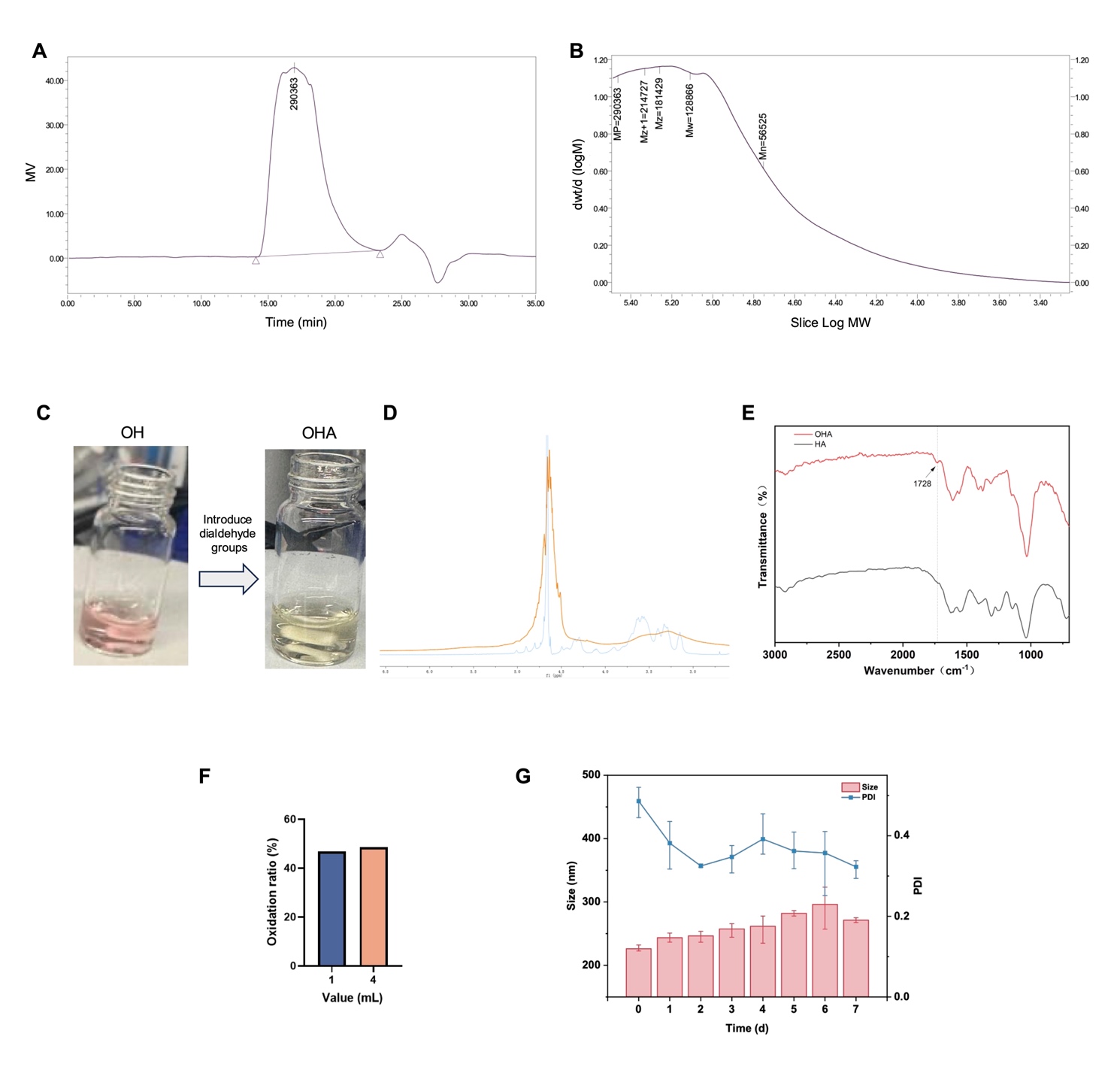
Figure S1 Characterization of HA Molecular Weight and Oxidation to OHA.** Supplemental to Figure 2.

**A-B.** Gel Permeation Chromatography (GPC) analysis of HA molecular weight. MV: Hydrodynamic Volume; MW: Molecular Weight; MP: Peak Molecular Weight; Mz: Z-Average Molecular Weight; Mz+1: Higher-Order Z-Average; Mn: Number-Average Molecular Weight. The mass distribution density per unit logarithmic molecular weight interval is represented by dwt/d(logM). **C.** Digital images illustrating the preparation of OHA via a ring-opening reaction of HA using sodium periodate. **D.** ^1^H NMR spectroscopy analysis of OHA, with peaks corresponding to the electronic environment around protons measured in parts per million (ppm). Newly formed peaks at 4.8 ppm and 4.9 ppm indicate successful oxidation. **E.** Fourier transform infrared (FT-IR) spectra of HA and OHA. The emergence of a characteristic peak at 1733 cm^-1^corresponding to the aldehyde (-C=O-) group in OHA. **F.** Quantification of the degree of oxidation in OHA prepared with 1 mL and 4 mL of reactant, determined via hydroxylamine hydrochloride titration. Data are presented as percentage oxidation. **G.** Serum stability of the PFTBA nanoemulsion.


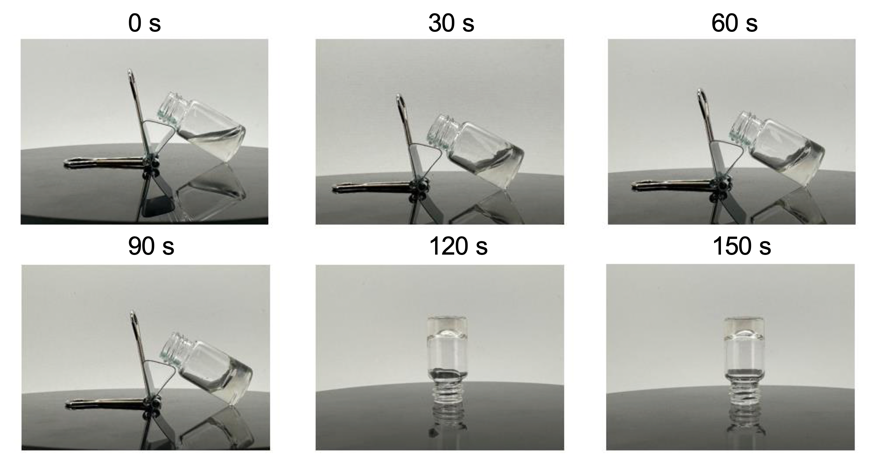


**Figure S2 Gelation time progression at 0 s, 30 s, 60 s, 90 s, 120 s, and 150 s of VOCP hydrogel.** Supplemental to Figure 2.


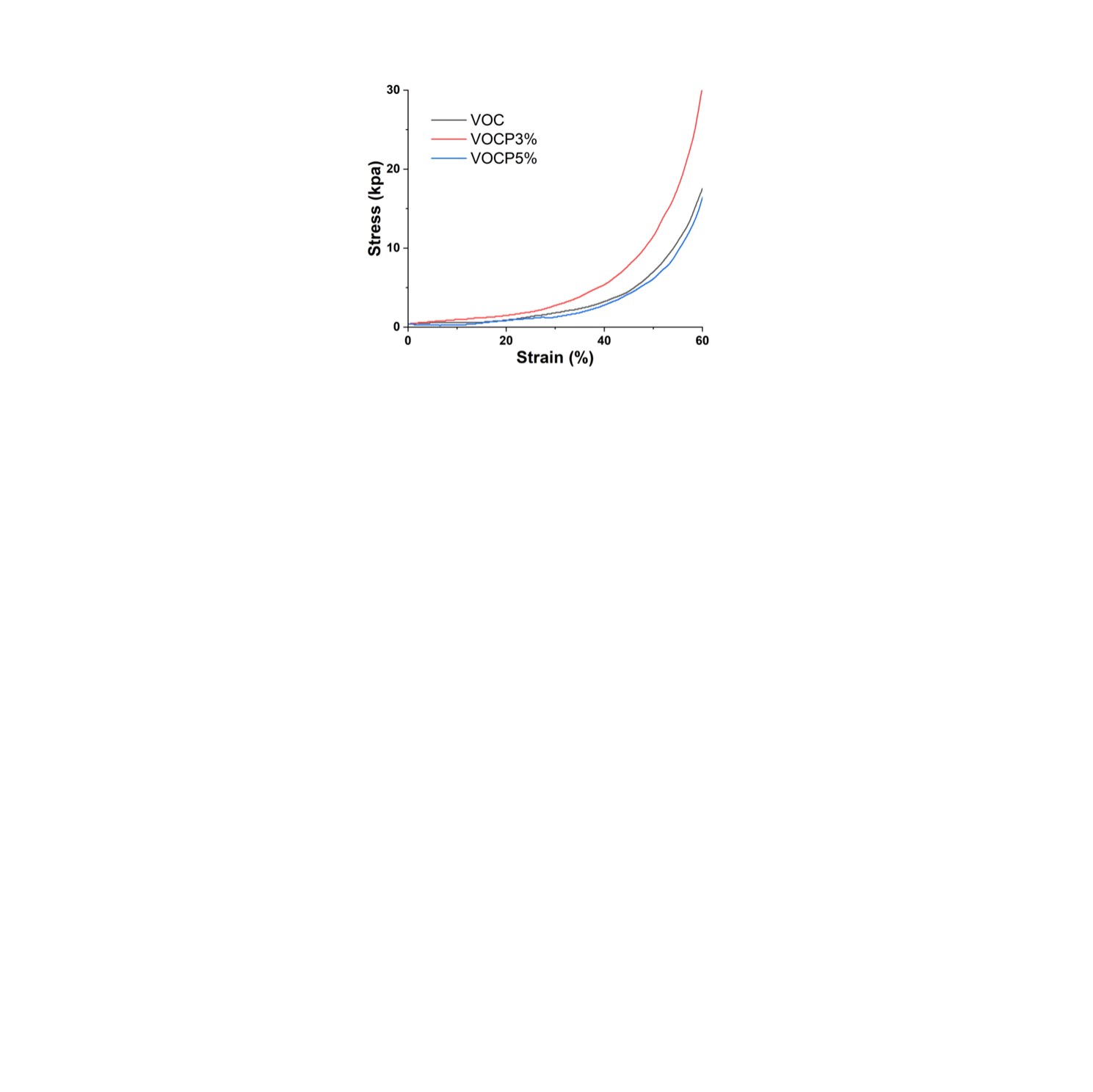


**Figure S3 Compression properties of VOC, 3% VOCP, 5% VOCP.** Supplemental to Figure 4.


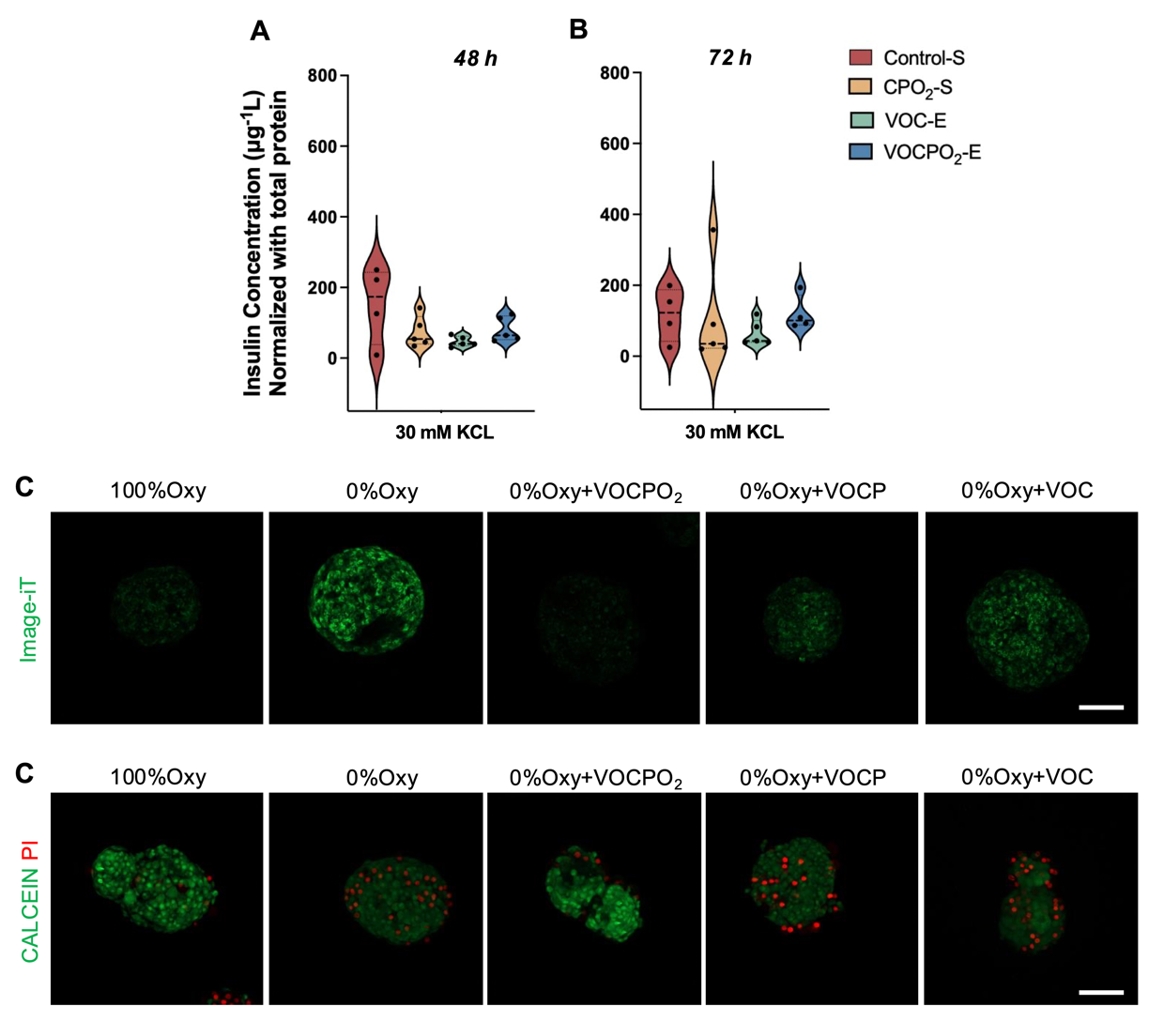


**Figure S4 Assessment of insulin secretion, hypoxia, and cell viability in mouse islets under various culture conditions.** Supplemental to Figure 6.

**A-B.** Glucose-stimulated insulin secretion (GSIS) was evaluated in mouse islets cultured under various conditions: suspension in culture media (Control-S), suspension in CMC-PFTBAO_2_ (CPO_2_-S), or encapsulation in VEGF+OHA+CMC hydrogel (VOC-E) or VEGF+OHA+CMC+PFTBAO_2_hydrogel (VOCPO_2_-E) after 48 hours **(A)** and 72 hours **(B)**. Insulin secretion was measured in media collected under low glucose (2.8 mM), high glucose (16.8 mM), and 30 mM KCl conditions. Insulin concentrations were determined via ELISA, normalized to total protein content, and expressed as µg/L. Statistical analysis was performed using one-way ANOVA. Data are presented as mean ± SD, with statistical significance denoted as *P < 0.05, **P < 0.01, ***P < 0.001, and ****P < 0.0001. **C.** Confocal images of mouse islets cultured under normoxia (100% O_2_) or hypoxia (0% O_2_) for 6 hours. Hypoxia groups included unencapsulated islets and islets encapsulated with VEGF+OHA+CMC+PFTBAO_2_ (VOCPO_2_), VEGF+OHA+CMC+PFTBA (VOCP), or VEGF+OHA+CMC (VOC). Hypoxia levels were assessed using the fluorogenic compound Image-iT, which fluoresces under decreased oxygen concentrations. Scale bar: 50 µm. **D.** Live/dead confocal imaging of mouse islets cultured under varying oxygen conditions for 6 hours. Live cells were visualized using calcein green (CALCEIN), and dead cells were labeled with propidium iodide (PI) red. Scale bar: 50 µm.


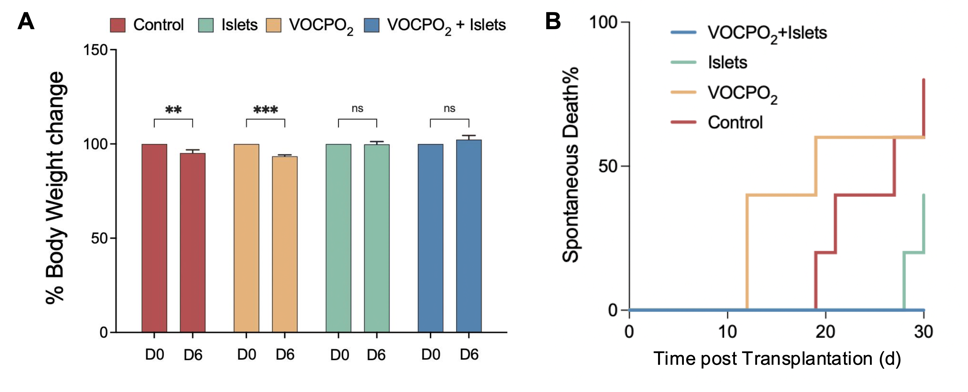


**Figure S5 VOCPO₂-encapsulated islets preserve body weight and enhance survival in STZ-induced diabetic C57BL/6J mice.** Supplemental to Figure 8.

**A.** Body weight changes over six days post-transplantation (n = 5 per group), with values on Day 6 (D6) normalized to Day 0 (D0). Data are presented as mean ± SEM and analyzed using one-way or two-way ANOVA. Statistical significance: ns, not significant (P > 0.05); **P < 0.01; ***P < 0.001, compared to the control group. **B.** Survival analysis showing the incidence of spontaneous death over one month post-transplantation.
